# Single-cell transcriptomic analysis reveals age-related remodeling of brain endothelial cells

**DOI:** 10.1016/j.nbas.2026.100164

**Published:** 2026-07-31

**Authors:** Hai Duc Nguyen, Summer Siddiqui, Diana G. Bohannon, Robert V. Blair, Hong-Wen Deng, Alexandre Prat, Woong-Ki Kim

**Affiliations:** a Division of Microbiology, Tulane National Biomedical Research Center, Tulane University, Covington, Louisiana, USA; b Department of Microbiology and Molecular Cell Biology, Eastern Virginia Medical School, Norfolk, Virginia, USA; c Division of Comparative Pathology, Tulane National Biomedical Research Center, Tulane University, Covington, Louisiana, USA; d Center for Biomedical Informatics & Genomics, John W. Deming Department of Medicine, Tulane University School of Medicine, New Orleans, Louisiana, USA; e Department of Neuroscience and CRCHUM, Faculty of Medicine, Université de Montréal, Montréal, Quebec, Canada; f Department of Microbiology & Immunology, Tulane University School of Medicine, New Orleans, Louisiana, USA

**Keywords:** Aging, Alzheimer's disease, Endothelial cell subtypes, Ramp2, Rasgrf2, scRNA-seq

## Abstract

Blood–brain barrier (BBB) integrity naturally declines with age. Brain endothelial cells (ECs) and pericytes (PCs) form the BBB, and aging impairs tight junctions, likely via altered PC-to-EC signaling. However, the molecular mechanisms underlying this impairment remain unclear. Using single-cell RNA sequencing, we profiled 68,316 brain ECs expressing 15,564 genes from young and old mice. Unsupervised clustering and annotation revealed five distinct EC subtypes—Capillary EC1, Capillary EC2, Arterial EC, Venous EC1, and Venous EC2—defined by marker genes *Mfsd2a, Plvap, Bmx, Nr2f2*, and *Vcam1*, respectively. Aging shifted EC subtype distribution, with reduced Capillary EC1 (45% vs. 57%) and increased Arterial (33% vs. 16%) and Venous ECs (12% vs. 2%) compared with young mice. Mio analysis further showed that Capillary EC1 and Venous EC2 neighborhoods were less abundant in aged brains. Biotin metabolism was decreased in old vs. young mice, particularly within Capillary EC1, Capillary EC2, and Arterial EC. Although widespread gene downregulation was observed across EC subsets, overall expression trends were largely consistent among clusters. Key genes—*Ramp2, Hbb-bs, Ly6c1, Calm1*—were less abundant, whereas *Rasgrf2* was uniquely enriched in aged mice. Immunohistochemistry confirmed reduced LY6C and RAMP2 and elevated RASGRF2 in aged mouse and human brains. Cell–cell interaction analyses revealed age-associated remodeling of ligand-receptor signaling. Enrichment analyses implicated pathways involved in neurovascular integrity, inflammation, amyloid processing, and vascular remodeling. Collectively, these findings show that aging reprograms EC subtype composition, gene expression, and metabolism, thereby contributing to BBB disruption and neurovascular dysfunction.

## 1 Introduction

Brain endothelial cells (ECs) play a crucial role in maintaining the integrity of the blood-brain barrier (BBB), serving as a selective interface that separates the brain from systemic circulation [1]. ECs are also key components of the neurovascular unit, working in close association with the extracellular matrix, pericytes (PCs), astrocytes, and neurons to regulate brain homeostasis [2]. Aging is related to impairing this unit, resulting in a reduced capacity to maintain stem cell and neuronal function, decreased cerebral blood flow, and increased toxic leakage [3], [4], [5]. Aging has also been strongly related to EC senescence, leading to functional deterioration of the BBB [6], [7]. Senescent ECs exhibit increased expression of inflammatory signaling pathways [e.g., nuclear factor κ B, interleukin 6 [8], and intercellular adhesion molecule 1 [9]], enhanced interactions with circulating immune cells [e.g., macrophages [10]], disruption of tight junction proteins [e.g., claudin-5 and occludin [11]], and impaired transcellular transport systems, including reductions in glucose transporter 1 (GLUT1) [12] and P-glycoprotein function [13], [14]. Together, these alterations contribute to BBB dysfunction and increased vulnerability of the aging brain to neurodegenerative processes.

The brain vasculature is highly heterogeneous, consisting of veins and venules, arteries and arterioles, and capillaries, each with unique molecular signatures and specialized functions [15]. Currently, advances in single-RNA sequencing (scRNA-seq) have made it possible to explore cellular heterogeneity in the brain ECs, revealing distinct gene expression profiles for EC subtypes in disease models such as autoimmune encephalomyelitis, multiple sclerosis, vascular diseases, hypertrophic scars, pulmonary arterial hypertension, and obesity [16], [17], [18], [19], [20].

Recent scRNA-seq studies have revealed that brain ECs show segment-specific gene expression patterns, with venous, arterial, and capillary ECs each playing different molecular roles. Among these, hippocampal capillary ECs appear particularly sensitive to aging, exhibiting pronounced transcriptional changes such as increased gene expression involved in oxidative stress responses (*Hsp90ab1*, *Hsp90aa1*) and innate immunity (e.g., *Nfkbia*, *Ifitm3*) [21], [22], [23]. By contrast, arterial and venous ECs show more modest transcriptional changes, with fewer differentially expressed genes (DEGs) related to these pathways, highlighting the unique vulnerability of capillary ECs to age-related molecular alterations [21]. Aging induces zonation-dependent transcriptomic changes in ECs, particularly in capillaries, affecting BBB integrity, energy metabolism, and immune signaling, some of which are associated with Alzheimer's disease (AD) risk genes and are pharmacologically reversible [24]. In addition, ECs display regional heterogeneity across the CNS, with specialized genes such as *Stra6* regulating local retinoid deposition and influencing the function of specific neural circuits [25].

Although previous scRNA-seq studies have provided valuable insights into the role of brain ECs in aging, they have mostly focused on broad vascular changes or the overall EC population, without resolving the transcriptomic profiles of individual EC subtypes [21], [22], [23]. In particular, these studies did not examine changes in EC subtype composition, gene expression dynamics, intracellular abundance, or metabolic pathways across subtypes. As a result, a comprehensive and systematic analysis of the transcriptomic landscape of all brain EC subtypes during aging is still lacking, leaving important gaps in our understanding of how aging selectively affects EC function and contributes to neurovascular decline.

To address this gap, we performed scRNA-seq to examine transcriptomic differences in brain ECs between young (14-week-old) and old (84-week-old) *C57BL/6* *J* mice. We focused on the heterogeneity of EC subtypes—arterial, capillary, and venous—to identify age-related changes in gene expression, subtype composition, inter-subtype communication, intracellular abundance, metabolic programs, and pathways that may contribute to endothelial dysfunction and neurovascular decline. By leveraging an unbiased single-cell approach, our study provides a detailed view of how aging reshapes the molecular and cellular landscape of brain ECs, offering new insights into the mechanisms underlying BBB dysfunction and its impact on brain health.

## 2 Materials and methods

### 2.1 Animals and tissue collection

All animal experiments were approved by the Institutional Animal Care and Use Committee (IACUC) of Eastern Virginia Medical School and conducted in accordance with NIH guidelines, as well as the ARRIVE 2.0 reporting standards. Male *C57BL/6* *J* mice were purchased from a commercial vendor and maintained under standard laboratory conditions with free access to food and water. Young male mice (14 weeks old, *n* = 3) and old male mice (84 weeks old, n = 3) were used in this study (Table 1). To reduce technical variability, all samples were collected and processed simultaneously by the same technician. In addition to single-cell mouse groups, separate groups of young and old mice (4M:4F), young and old rhesus macaques (*Macaca mulatta*, RMs) (6M:6F), and young and old humans (5M:4F) were used for IHC validation (Table 1). For RMs, formalin-fixed paraffin-embedded (FFPE) sections of the frontal white matter were obtained from young and aged RMs housed at the Tulane National Biomedical Research Center (TNBRC). Animal procedures were approved by the Tulane University Institutional Animal Care and Use Committee and performed in accordance with the NIH Guide for the Care and Use of Laboratory Animals and the Weatherall Report. Human FFPE sections of frontal white matter from young and aged individuals were kindly provided by the Manhattan HIV Brain Bank (MHBB) and NIH NeuroBioBank. These samples exhibited only minimal, non-diagnostic abnormalities on autopsy, and all postmortem intervals were less than 48 h (Table 1).

**Table 1:** Samples used in this study for single cell RNA sequencing and immunohistochemistry.

**No**	**ID**	**Brain area**	**Species**	**Age**	**Gender**	**Group**
**For single cell RNA sequencing**						
1.	Parse Young	Whole brain	C57BL/6 Mouse	14.0-week	Male	Young
2.	Parse Young	Whole brain	C57BL/6 Mouse	14.0-week	Male	Young
3.	Parse Young	Whole brain	C57BL/6 Mouse	14.0-week	Male	Young
4.	Parse-Aged	Whole brain	C57BL/6 Mouse	84.0-week	Male	Old
5.	Parse-Aged	Whole brain	C57BL/6 Mouse	84.0-week	Male	Old
6.	Parse-Aged	Whole brain	C57BL/6 Mouse	84.0-week	Male	Old

**For immunohistochemistry**						
7.	8619 (nSxYAF2)	Whole brain	C57BL/6 Mouse	14.0-week	Female	Young
8.	8620 (nSxYAF1)	Whole brain	C57BL/6 Mouse	14.0-week	Female	Young
9.	8628 (nSxYAM1)	Whole brain	C57BL/6 Mouse	14.0-week	Male	Young
10.	8629 (nSxYAM2)	Whole brain	C57BL/6 Mouse	14.0-week	Male	Young
11.	8286 (nSxAAF2)	Whole brain	C57BL/6 Mouse	57.0-week	Female	Old
12.	8287 (nSxAAF1)	Whole brain	C57BL/6 Mouse	57.0-week	Female	Old
13.	8290 (nSxAAM1)	Whole brain	C57BL/6 Mouse	57.0-week	Male	Old
14.	8291 (nSxAAM2)	Whole brain	C57BL/6 Mouse	57.0-week	Male	Old
15.	5958	Frontal cortex	Human	22.0-yr	Male	Young
16.	6061	Frontal cortex	Human	24.0-yr	Male	Young
17.	6169	Frontal cortex	Human	25.0-yr	Male	Young
18.	6235	Frontal cortex	Human	21.0-yr	Female	Young
19.	HCT17HEV	Frontal cortex	Human	30.0-yr	Female	Young
20.	HCtZA	Frontal cortex	Human	79.0-yr	Female	Old
21.	3517	Frontal cortex	Human	82.0-yr	Male	Old
22.	5171	Frontal cortex	Human	79.0-yr	Male	Old
23.	5511	Frontal cortex	Human	80.0-yr	Female	Old
24.	GI53 / 11A313	Frontal cortex	*Macaca mulatta*	5.02-yr	Male	Young
25.	GI84 / 11A635	Frontal cortex	*Macaca mulatta*	5.14-yr	Male	Young
26.	IT02 / 11A023	Frontal cortex	*Macaca mulatta*	4.69-yr	Male	Young
27.	IT24 / 11A014	Frontal cortex	*Macaca mulatta*	4.77-yr	Male	Young
28.	JC51 / 14A325	Frontal cortex	*Macaca mulatta*	4.09-yr	Female	Young
29.	JE71 / 14A333	Frontal cortex	*Macaca mulatta*	4.03-yr	Female	Young
30.	CJ25 / 16A423	Frontal cortex	*Macaca mulatta*	17.77-yr	Female	Old
31.	CM76 / 17A381	Frontal cortex	*Macaca mulatta*	17.12-yr	Female	Old
32.	P346 / 16A070	Frontal cortex	*Macaca mulatta*	22.80-yr	Female	Old
33.	213	Frontal cortex	*Macaca mulatta*	23.00-yr	Male	Old
34.	208	Frontal cortex	*Macaca mulatta*	25.00-yr	Male	Old
35.	352	Frontal cortex	*Macaca mulatta*	26.00-yr	Male	Old

### 2.2 Single-cell RNA sequencing tissue processing

Phosphate-buffered saline (PBS) perfused brains were collected from six mice (Table 1). Each brain was cut into ∼10 sagittal slices and dissociated using the adult mouse brain dissociation kit (Miltenyi 130‐107-677) on a gentleMACS™ Octo Dissociator with heaters. Cell suspensions underwent debris removal and red blood cell lysis, followed by labeling with streptavidin-conjugated antibodies against CD11b, O4, and ACSA-2 for 20 min, washing twice, and incubation with biotinylated magnetic beads for 15 min. Flowthrough, enriched for vascular cells, was collected while immune cells, oligodendrocytes, and astrocytes were retained in the column. To reduce neuronal contamination, the vascular-enriched fraction was subjected to a freeze-thaw cycle (1 °C/min to −80 °C overnight, then rapidly thawed at 37 °C), preferentially killing neurons. Dead cells were removed using Annexin V dead cell removal magnetic separation (Miltenyi). The remaining fraction was processed using Parse Biosciences WT 100 k for fixation, barcoding, and library preparation per manufacturer instructions. Libraries were sequenced on a NovaSeq 6000 targeting 100,000 cells per sample, with an average depth of 50,000 reads per cell (∼5 billion total reads).

### 2.3 Data processing and quality control

Raw sequencing data were demultiplexed and aligned to the *mm10 mouse* genome using CellRanger (10× Genomics, v6.0) per Parse's recommendations. scRNA-seq was performed using Scanpy (v1.9.1) in Python (v3.12.3). First, quality control metrics were assessed using Scanpy's pp.calculate_qc_metrics, including total counts, number of expressed genes, and mitochondrial gene percentage. Ribosomal genes were removed to avoid obscuring biological signals. Cells with >25% mitochondrial content (indicative of stress/death) were excluded. We also excluded cells expressed with low library; in this study, genes expressed in <200 cells were excluded based on the distribution of libraries in our datasets.

### 2.4 Dimensionality reduction and clustering

Highly variable genes (HVGs) were identified using scanpy.sc.pp.highly_variable_genes (Seurat v3 flavor), selecting the top 4000 genes from raw counts (layer = ‘raw_counts’). Principal component analysis (PCA) was run with sc.tl.pca (100 components), and the top 30 PCs (adata_clean.obsm[‘X_pca’][:,0:30]) were used to build a nearest-neighbor graph (sc.pp.neighbors, 30 neighbors, Euclidean distance, use_rep = ‘X_pca’). Uniform Manifold Approximation and Projection (UMAP; sc.tl.umap, min_dist = 0.1) and force-directed layout (FDL; sc.tl.draw_graph, layout = ‘fa’) were used for visualization. Cells were clustered with Leiden (sc.tl.leiden, resolution = 1) and PhenoGraph (sc.external.tl.phenograph, k = 30, Jaccard metric, resolution = 0.5); the Jaccard similarity graph was stored as a sparse CSR matrix for efficiency. UMAP plots comparing Leiden and PhenoGraph results were generated with sc.pl.umap.

### 2.5 Cell type annotation

For cell population characterization, we used highly variable genes for dimensionality reduction via PCA, constructed a nearest-neighbor graph, and applied UMAP for visualization. Cell clustering was performed using PhenoGraph, an unsupervised algorithm designed to identify phenotypically distinct cell populations in high-dimensional single-cell data. Annotation was performed with CellTypist (v1.6.0) using the Mouse Whole Brain model (Mouse_Whole_Brain.pkl). Data were normalized to 10,000 counts per cell (sc.pp.normalize_total) and log-transformed (sc.pp.log1p). UMAP coordinates were transferred from the primary dataset, and annotations were manually cross-validated with published datasets [26], [27], [28], [29]. ECs, labeled “333 Endo NN,” were confirmed by strong expression of *Pecam1*, *Cdh5*, and *Slc2a1*. Five EC subtypes were defined based on high-confidence marker genes [30]: capillary (Cap) EC1 (*Mfsd2a, Cldn5, Slc2a1*), enriched for tight junction gene expression [31]; Cap EC2 (*Plvap, Igfbp7, Car4*), enriched for vascular permeability; arterial (Art) EC (*Bmx, Efnb2, Gja5, Dll4, Sox17*), enriched for Tie2, VEGFR1, and canonical signaling in arterial ECs [32]; venous (Ven) EC1 (*Nr2f2*, *Ephb4, Ackr1, Nrp2, Emcn*), representing baseline venous lineage identity; and Ven EC2 (*Vcam1, Sele, Selp*), enriched for inflammation/adhesion markers [33]. Pan-endothelial markers (*Pecam1, Cdh5*) were used to confirm endothelial identity across clusters, and lymphatic markers (*Prox1, Lyve1, Pdpn*) were examined to exclude lymphatic contamination. SEACells is a graph-based method to define metacells—groups of cells representing distinct, fine-grained states, where most variation within each group is technical rather than biological. To further validate cell identity, we applied SEACells, an algorithm that aggregates transcriptionally similar single cells into “metacells.” Metacells are groups of transcriptionally similar single cells that exhibit consistent gene expression patterns, thereby reducing noise and revealing stable cellular states [34]. Over 95% of these metacells mapped to EC populations, confirming the robustness of the annotation.

### 2.6 Doublet detection and cell cycle analysis

Doublets were detected using Scrublet (v0.2.3) with the following parameters: expected_doublet_rate = 0.06, sim_doublet_ratio = 2.0, knn_dist_metric = ‘euclidean’, log_transform = True, n_prin_comps = 30, and random_state = 0 [35]. Clusters with unusually high mean doublet scores were flagged and removed when they appeared as clear outliers. Next, cell cycle scores were calculated using tl.score_genes_cell_cycle in Scanpy, based on established G1 phase, S-phase, and G2/M gene sets, to evaluate potential differences in cell cycle composition between animals [36].

### 2.7 Differential expression and enrichment analysis

Differential expression analysis was performed for each EC subtype using DESeq2 (v1.32.0) integrated via Scanpy (v1.9.1) on pseudobulk profiles aggregated per biological replicate. DEGs were defined as those with adjusted *p* < 0.05 and |log₂FC| ≥ 1. Visualization included heatmaps for subtype-specific expression patterns and volcano plots generated with EnhancedVolcano (*p*Cutoff = 10^−6^, FCcutoff = 0.5) in R (v4.1.2). Pathway enrichment analysis and protein-protein interaction analysis were performed using Enrichr, leveraging ranked gene lists from scanpy.tl.rank_genes_groups (using *GO_Biological_Process_2024* gene set for mice) and Metascape, respectively. Network interactions between these DEGs were generated by String (v12.0). We next examined interactions between DEGs from EC subtypes, microRNAs (miRNAs), and transcription factors (TFs). Associations between DEGs and miRNAs were analyzed using MIENTURNET [34], and miRNAs were considered significant if they showed an adjusted *p*-value (*FDR*) ≤ 0.05 and were highly connected to multiple genes. Links between DEGs and TFs were identified using *CHEA3* [37]*.* Key transcription factors were defined based on higher mean rank scores and greater overlap with the identified DEGs.

### 2.8 Gene–gene interaction and cross-study comparison analysis

In this section, we identified DEGs within each EC subtype using DESeq2. We then combined DEGs across all subtypes, retaining genes that were significantly enriched in at least one subtype (adjusted *p*-value < 0.05, |log₂FC| ≥ 1). This resulted in a set of 103 DEGs, which were used for gene–gene interaction analysis via GeneMANIA (https://genemania.org/). The resulting interaction network was used to estimate relationships among DEGs, including co-expression, physical interaction, and pathway co-membership. To support our findings, we compared EC subtype markers identified in the current dataset with those from a previously published mouse brain endothelial dataset (GEO accession: GSE147693). We selected this dataset as it analyzed comparable brain vascular samples from young (2–3 months) and old (18–20 months) CS57BL/6 J mice using the same dissociation and single cell isolation protocols [38].

Differential expression profiles were conducted using scanpy.sc.tl.rank_genes_groups function with the Wilcoxon rank-sum test. Differential expression profiles were considered markers if they met the criteria of a false discovery rate (FDR)-adjusted *p*-value < 0.05 and an absolute log2 fold change (|log2FC|) > 0.25. Shared differential expression profiles between datasets were identified by intersecting these gene lists. The similarity of log2FC between the two datasets was evaluated using the Pearson correlation coefficient (r). Genes showing the same direction of change (enrichment in either young or old samples) across studies were further examined. To investigate the biological relevance of these shared differential expression profiles, we manually curated their reported roles from PubMed, GeneCards, and UniProt databases, followed by pathway categorization into processes related to angiogenesis, endothelial function, inflammation, immune response, cell–cell adhesion, stress response, BBB function, and molecular transport.

### 2.9 Intracellular abundance of mRNA in endothelial cell neighborhoods

To assess the differential abundance of EC populations between young and old mice, we applied Milo analysis using the miloR package (v1.0.0) on a normalized, log-transformed scRNA-seq dataset with PCA embeddings [39]. A k-nearest neighbor graph (k = 30, d = 30) was constructed using buildGraph, followed by neighborhood definition (makeNhoods, prop = 0.1) and cell counting per sample (countCells) across EC subtypes (Cap EC1, Cap EC2, Art EC, Ven EC1, Ven EC2). Differential abundance was tested with testNhoods using a design matrix specifying condition (old vs. young) and sample replicates, with spatial FDR correction applied via calcNhoodDistance. Average expression of neighborhood graphs, colored by log fold change, was visualized for neighborhoods with SpatialFDR <0.05 to assess enriched EC neighborhoods in old and young mice.

### 2.10 Cell-cell communication

To assess ligand-receptor interactions between ECs and other related cell types (smooth muscle cells (SMCs), pericytes (PCs), astrocytes (ACs), and vascular and leptomeningeal cells (VLMCs) in old mice, we used LIANA's CellPhoneDB method (v0.1.0) on our normalized dataset with the mouseconsensus resource [40]. Ligand-receptor interactions were inferred using LIANA (CellPhoneDB method) based on cluster-level gene expression and permutation testing. Because this approach does not inherently adjust for differences in cell abundance between conditions (EC numbers differed between young and old mice), interaction counts were additionally normalized to the total number of endothelial cells within each age group to estimate communication density. Significant interactions (*p* ≤ 0.01) between source ECs, EC subtypes, SMCs, PCs, ACs, and VLMCs were identified, visualized using line graphs, dot plots, and a chord diagram. Pathway enrichment analysis, including biological process, cellular component, and disease, was performed using ToppGene Suite [41]. Venn diagrams were generated to identify shared ligands and shared receptors between ECs, EC subsets, SMCs, PCs, ACs, and VLMCs, with their roles in amyloid processing, neuroinflammation, and BBB integrity annotated via literature review.

### 2.11 Gene trend analysis

To assess gene change in ECs of young and old mice, we inferred pseudotime trajectories using Palantir (v1.0.0) on our normalized dataset to compute multiscale diffusion maps (n_eigs = 8) [42]. We generated dot plots in Scanpy for selected pathways relevant to BBB integrity and endothelial function to visualize gene expression differences between young and old ECs. These included CSF1R signaling (*Csf1*), which regulates microglial activation and endothelial homeostasis [43], and lipid metabolism (*Apoe*), which plays a critical role in BBB maintenance and vascular health [44]. We also examined key EC-related genes (*Ramp2, Hbb-bs, Rasgrf2, Ly6c1, Calm1*), which were commonly expressed across multiple EC subtypes. Data were subset to different clusters 0, 1, 2, and 3 based on PhenoGraph clustering (sc.external.tl.phenograph; k = 30, Jaccard metric, resolution = 0.5), grouped by condition (old vs. young), and plotted to compare expression patterns across groups.

To identify age-associated transcriptional changes, we performed different expression analyses between ECs from young and old mice. We used the Wilcoxon rank-sum test implemented in Scanpy (sc.tl.rank_genes_groups) to compare the old group against the young reference group. The top-ranked DEGs were visualized using sc.pl.rank_genes_groups. To confirm the gene trend patterns in our dataset, gene expression trends for 1000 randomly sampled genes were visualized along Branch1 using plot_gene_trends and clustered with cluster_gene_trends, with MAGIC imputation (*t* = 3, PCA-based, number of components = 30, metric = euclidean) [45]. Furthermore, gene expression trends for key of interest genes (*Ramp2, Hbb-bs, Rasgrf2*), which are broadly expressed across multiple EC subtypes, were visualized along five branches using plot gene trends and clustered with cluster gene trends, with MAGIC imputation (t = 3, PCA-based) to enhance trend clarity.

### 2.12 Biotin metabolism factor analysis

To explore co-varying gene programs in ECs, we applied factor analysis using the SPECTRA package (v0.1.0) on our dataset to identify metabolic states across EC subtypes [46]. In this study, we focused on biotin metabolism because it plays a critical role in endothelial energy balance, redox regulation, and BBB integrity [47], [48]. Predefined gene sets included subtype-specific markers (e.g., *Mfsd2a* for Cap EC1, *Bmx* for Art EC, *Nr2f2* for Ven EC1, *Vcam1* for Ven EC2, *Plvap* for Cap EC2) and global processes (biotin metabolism: *Slc16a1, Btd, Slc5a6, Slc19a3*) [49], [50]. The analysis used highly variable genes, cell-type-specific factors (use_cell_types = True), and parameters lam = 0.1, rho = 0.001, and 100 epochs, with an overlap threshold of 0.2 to ensure distinct factors.

### 2.13 Immunohistochemistry

To confirm our observations in a translationally relevant in vivo context, we carried out IHC on brain tissues from mice in a separate cohort, Indian RMs, and humans. FFPE sections were incubated at 58–60 °C for 1 h or overnight, deparaffinized in xylene (2 × 5 min), and rehydrated through graded ethanol (100%, 95%, 70%; 2 × 5 min each) before rinsing in distilled water. Antigen retrieval was performed in citrate-based Antigen Unmasking Solution (Vector Laboratories, H-3300) by microwaving for 20 min, followed by a 20 min cooldown at room temperature. Sections were washed in TBS-T (0.05% Tween-20; 2 × 5 min), treated with BLOXALL (Vector Laboratories) for 10 min to block endogenous peroxidase, and incubated with 5% normal goat serum (NGS) in TBS-T (0.05% Tween-20) for 30 min at room temperature. Sections were incubated for 1 h with rabbit polyclonal antibodies against LY6C (#ab314120, Abcam), RAMP2 (#sc-365240, Santa Cruz), or RASGRF2 (#ab121577, Abcam), diluted 1:5–1:50 in Dako antibody diluent (Table 2). For RAMP2 staining in mouse sections, the Mouse on Mouse (M.O.M.) Kit (Vector Laboratories, #BMK-2202) was used before primary antibody incubation to reduce endogenous mouse IgG staining, following the manufacturer's instructions. Sections were incubated with a biotinylated goat anti-rabbit IgG secondary antibody (Vector Laboratories, BA-1000; 1:200) or the secondary antibody provided in the M.O.M. Kit for 30 min at room temperature, followed by Vectastain Elite ABC reagent (Vector Laboratories, SK-7100; 30 min). Signals were developed using ImmPACT DAB (Vector Laboratories, SK-4105; 2–5 min), and slides were counterstained with Mayer's hematoxylin (Dako, S3309), rinsed, dehydrated in graded ethanol/xylene, and mounted with VectaMount (Vector Laboratories, H-5000). Images were captured with a Zeiss Axio Scan.Z1 and analyzed using HALO HighPlex FL v4.1.3 (Indica Labs) from 20 sections, including the hippocampal region in mice. The DAB-positive area (brown chromogen) and hematoxylin-stained nuclei (blue) were identified based on color deconvolution, and thresholds were manually adjusted to ensure accurate detection of specific staining. This normalized value was then used for comparisons between young and old groups. Protein–protein interaction and pathway enrichment analyses for RAMP2 and RASGRF2 were performed using the STRING database (v12.0) with default settings. Functional enrichment for Gene Ontology (GO), KEGG, and WikiPathways terms was conducted based on the STRING network output.

**Table 2:** Antibodies used in this study.

Antigen	Catalogue number	Host	Isotype	Reactivity	Manufacturer	IHC Conc.
RAMP2	#sc-365240	Mouse	Mouse mAb_IgG2a kappa	Human, mouse, rat	Santa Cruz	1:10,000
LY6C	#ab314120	Rabit	Rabbit pAb_IgG	Human	Abcam	1:200
RASGRF2	#ab121577	Rabit	Rabbit mAb_IgG	Mouse	Abcam	1:500

### 2.14 Statistical analysis

All analyses were conducted in Python 3.12.3 with Scanpy as the core framework and R v4.1.2 integrated via Scanpy. Wilcoxon rank-sum tests were used for gene ranking, and *p*-values were adjusted for multiple testing (Benjamini-Hochberg). Data are presented as means ± SEM; significance was set at *p* < 0.05. Violin plots were generated using ggpubr and ggplot2, with significance levels annotated. A heatmap was generated using the pheatmap package in R based on the percentage of DAB-positive signal per marker (RAMP2, LY6C, and RASGRF2) per dataset.

## 3 Results

### 3.1 Dataset characteristics

To investigate transcriptomic differences in brain ECs between young and old mice, we performed scRNA-seq on brain tissue from young (14-week-old, *n* = 3 males) and old (84-week-old, n = 3 males) C57BL/6 J mice. Our dataset initially comprised 68,316 cells and 17,821 genes. As shown in Fig. S1, no outliers in the doublet score were observed in the dataset. After filtering, we retained a high-quality dataset of 68,316 cells and 15,564 genes for downstream analysis. Cell cycle status can influence scRNA-seq data in both young and old mice. In young mice, it reflects normal proliferation, while in older mice, it often signals disrupted cycling linked to aging [51], [52]. To deconvolute cell cycle effects and age-related changes, we assessed the cell cycle distribution of ECs from young and old mice. We found no significant differences likely to affect downstream analysis (Fig. S2A—C). Finally, our data included 5426 cells (15,435 genes) from old mice (Old 1 = 1752 cells, 15,408 genes; Old 2 = 1718 cells, 15,441 genes; Old 3 = 1956 cells, 15,456 genes) and 62,890 cells (15,564 genes) from young mice (Young 1 = 31,315 cells, 15,564 genes; Young 2 = 9137 cells, 15,564 genes; Young 3 = 22,438 cells, 15,564 genes).

**Fig. 1:**
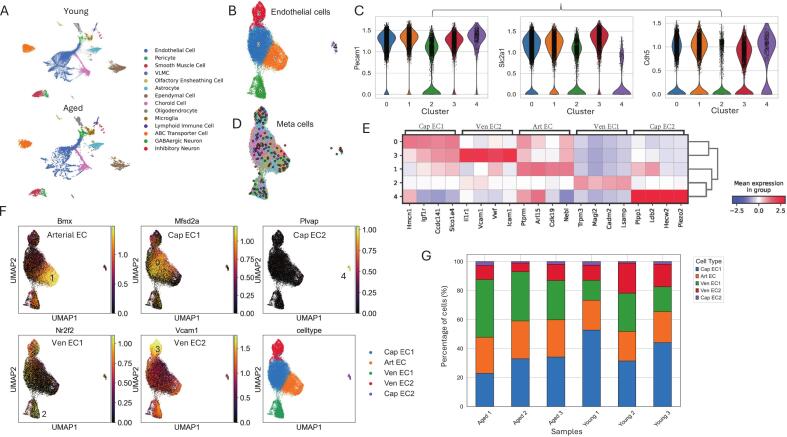
Identification and characterization of brain endothelial cell subtypes in young and old mice. (A) UMAP visualization of 68,316 cells from young (14-week-old, *n* = 3) and old (84-week-old, n = 3) C57BL/6 J mouse brains, clustered using PhenoGraph, identifying 14 major cell populations. (B) UMAP visualization of 30,462 cells from the EC cluster “333 Endo NN”. (C) Violin plots showing high expression of canonical EC markers (*Pecam1*, *Cdh5*, *Slc2a1*) across EC clusters, confirming endothelial identity. The color scale represents log-transformed normalized gene expression levels. (D) SEACells metacell analysis validating the EC cluster, with >95% alignment and revealing fine-grained EC states. (E) Heatmap of top five marker genes per EC subtype, identified via Wilcoxon rank-sum test. (F) UMAP projection of EC subtype proportions (Capillary EC1: *Mfsd2a*, Capillary EC2: *Plvap*, Arterial EC: *Bmx*, Venous EC1: *Nr2f2*, Venous EC2: *Vcam1*). The color scale represents log-transformed normalized gene expression levels. (G) Stacked bar plot comparing EC subtype proportions between young and old mice.

We identified 14 major clusters representing ECs and other cell types (Fig. 1A). To focus on ECs, we selected the cluster labeled “Endothelial cell,” which contained 30,462 cells and 17,724 genes. Endothelial identity was confirmed by the high expression of canonical EC markers *Pecam1, Cdh5*, and *Slc2a1* across all EC clusters (Fig. 1B, C, and Fig. S3—A, B) [27]. To further validate our EC population, we applied SEACells, a graph-based approach that aggregates transcriptionally similar single cells into ‘metacells.’ This analysis showed that over 95% of the identified metacells aligned with our previously defined EC cluster, supporting the accuracy of our cell selection (Fig. S3C). The SEACells analysis also revealed a subset of metacells representing distinct, fine-grained EC states, further underscoring the heterogeneity within this population (Fig. 1D). Using the Wilcoxon rank-sum test, we calculated enrichment scores and ranked genes by their expression within each EC cluster. Fig. 1E highlights the top five marker genes for each of the five EC subtypes. To further refine EC subtype classification, we established marker genes: *Mfsd2a, Cldn5, Slc2a1* (Cap EC1), *Plvap, Igfbp7, Car4* (Cap EC2), *Bmx, Efnb2, Gja5, Dll4, Sox17* (Art EC), *Nr2f2, Ephb4, Ackr1, Nrp2, Emcn* (Ven EC1), and *Vcam1, Sele, Selp* (Fig. S3B) (Ven EC2) [30]. These markers robustly distinguished five EC subtypes, with the majority of cells belonging to Cap EC1 and Ven EC1 populations (Fig. 1F). Notably, the relative abundance of these subtypes differed between young and old mice (Fig. 1G).

### 3.2 Aging alters gene expression in brain endothelial cells

**Fig. 2:**
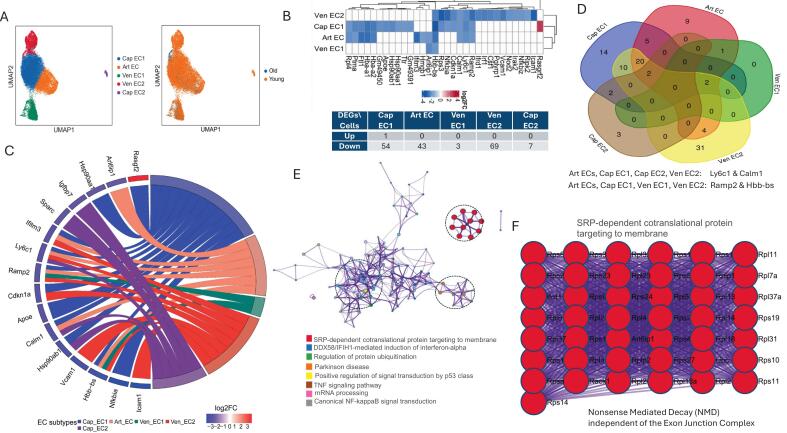
Differential gene expression and enrichment analysis in aging brain endothelial cells. (A) UMAP visualization of cell types and cells in young and old mice. (B—C) The top 25 differentially expressed genes (DEGs) shown in the heatmap and chord diagram highlight key depleted DEGs in old mice. (D) a Venn diagram of key DEGs (*Ramp2*, *Hbb-bs*, *Ly6c1*, *Calm1*) consistently depleted across four EC subtypes. (E) Enrichment analysis and protein-protein interaction analysis (F) in brain endothelial cells using Metacape. Selected pathways with p-adjusted < 0.05 are presented.

Fig. 2A shows UMAP plots of EC subtypes and single cells from young and old brain samples. To identify genes and molecular pathways associated with aging in ECs, we performed differential expression analysis on each EC subtype from the single-cell dataset using DESeq2 (Fig. S4). Notably, DEGs between young and old mice were almost exclusively enriched in young mice (e.g., *Nfkbia, Hsp90ab1, Calm1, Cdkn1a, Ramp2, Ly6c1, Ifitm3, Hsp90aa1, Apoe, Vcam1, Icam1, Arl6ip1, Igfbp7, Sparc, and Hbb-bs),* with only one notable gene enriched in old mice (*Rasgrf2*) (Fig. 2, B and C).

Using Venn diagram analysis, we identified several genes that were commonly enriched across multiple EC subtypes in mouse brains (Fig. 2D and Fig. S5A—D). For example, *Ramp2* and *Hbb-bs* were co-expressed in Art ECs, Cap EC1, Ven EC1, and Ven EC2 subsets, suggesting their broad involvement in vascular function and endothelial homeostasis [53]. In contrast, *Ly6c1* and *Calm1* were shared among Art ECs, Cap EC1, Cap EC2, and Ven EC2, indicating their potential roles in maintaining endothelial stability and intercellular communication. Collectively, the consistent enrichment of these genes across multiple EC subtypes in young mice highlights their importance in preserving neurovascular integrity and overall brain health during aging.

Using DEGs from these EC subtypes, we performed enrichment analysis. We found that pathways related to SRP-dependent cotranslational protein targeting to the membrane, canonical NF-κB signal transduction, TNF signaling, death receptor signaling, neurodegenerative disease processes, fluid shear stress and atherosclerosis, p53-mediated signal transduction, and regulation of translation (Fig. 2E, F, and Fig. S6—A, B). Collectively, these results suggest that aging suppresses genes critical for EC function while selectively inducing genes like *Rasgrf2*, a finding that may reflect adaptive or stress-related transcriptional responses but requires further functional investigation [54], [55].

We next gathered all DEGs across EC subtypes to assess the association between these DEGs, miRNAs, and transcription factors associated with aging and EC function. We found that *Calm1*, which encodes calmodulin 1—a calcium-binding protein critical for maintaining vascular endothelial health and repair [56]—was linked to *miR-1a-3p*. Meanwhile, *Cdkn1a* (encoding the protein p21), a well-known regulator of cellular senescence [57], was associated with *miR-294-3p*, *miR-295-3p, miR-302d-3p*, and *miR-291b-3p* (Fig. S6C). Additionally, many Cap EC1 DEGs were connected to transcription factors known to regulate aging and EC biology, including TP53, EGR1, ATF3, and MYC ([58], [59], [60]) (Fig. S6D). These miRNAs and transcription factors have previously been implicated in endothelial dysfunction and vascular aging, supporting their potential roles as key regulators of age-related changes in endothelial cell biology [61], [62], [63], [64], [65].

**Fig. 3:**
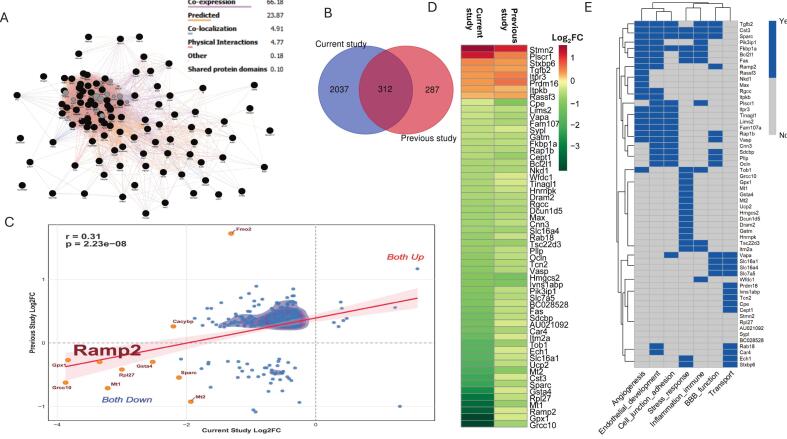
Cross-study validation and canonical functional characterization of endothelial cell (EC) aging-associated genes. (A) Gene–gene interaction network of 103 differentially expressed genes (DEGs) identified across EC subtypes generated using GeneMANIA. (B) Venn diagram showing the overlap of EC subtype markers between the current dataset and a previous mouse brain endothelial dataset (GEO accession: GSE147693). (C) Scatter plot comparing log2 fold changes (log2FC) of overlapping DEGs between the two datasets. (D) Bar plot summarizing shared markers with the same direction of expression change. (E) Functional categorization of shared markers based on literature and database searches (PubMed, GeneCards, UniProt, and aging- or BBB-specific databases).

We next assessed the gene–gene interaction network of 103 DEGs identified across all EC subtypes using GeneMANIA. The majority of these genes exhibited co-expression (66%), indicating that these genes work together and may take part in similar functions within ECs (Fig. 3A). To strengthen our findings, we compared EC subtype markers identified in the current study with those from a previous dataset (GEO accession: GSE147693) using differential expression analysis (sc.tl.rank_genes_groups, method = ‘wilcoxon’). Differential expression profiles were defined as genes with an FDR-adjusted *p-value* < 0.05 and an absolute log2FC (|log2FC|) > 0.25 (Table S1). We identified 312 shared markers between the two datasets (Fig. 3B). The overlap in differential expression profiles patterns was modest (*r* = 0.31, *p* < 0.001), which could be attributed to differences in cell isolation, library preparation, sequencing depth, or biological heterogeneity (e.g., mouse strain, brain region, or microenvironment) [24]. Consistent with its enrichment in young mice, *Ramp2* was found to be downregulated in a previous dataset, suggesting a potential decline in its vascular protective role with aging [53] (Fig. 3C). In total, 57 shared differential expression profiles exhibited the same direction of change between studies (8 upregulated and 49 downregulated; Fig. 3D and Table S2). We next searched the PubMed, GeneCards, UniProt databases to determine the canonical functions of these markers. The shared markers were enriched in pathways related to angiogenesis, endothelial function, inflammation, immune response, cell–cell adhesion, stress response, BBB function, and molecular transport (Table S3). The consistent downregulation of these genes in aged mice suggests that aging impairs endothelial function and compromises BBB integrity (Fig. 3E).

**Fig. 4:**
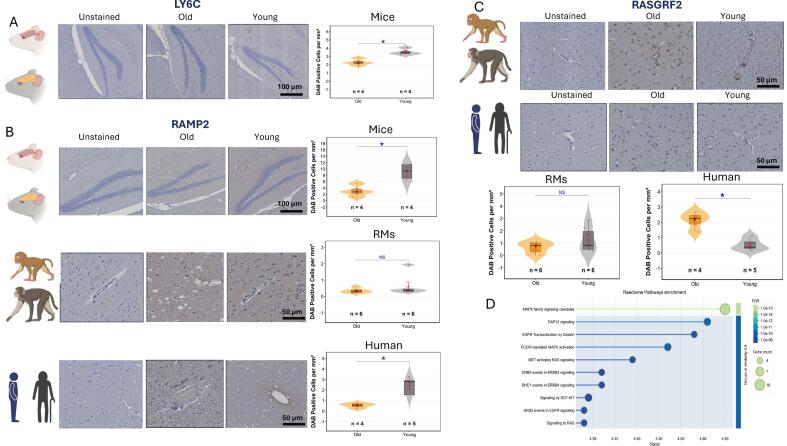
Protein expression of LY6C, RAMP2, and RASGRF2 in mouse, rhesus macaque, and human brain tissues. (A–C) Representative immunohistochemistry images showing protein levels of LY6C, RAMP2, and RASGRF2 in young and old mouse, rhesus macaque (RM), and human brain tissues. Quantification is presented as mean ± SEM (red) and median (blue cross). DAB-positive signal was normalized to tissue area using the equation: DAB Positive / Area (mm^2^). Wilcoxon rank-sum test; *, *p*-value <0.05; ns: non-significant. (D) STRING-based enrichment analysis of RASGRF2. Protein–protein interaction enrichment analysis was generated using STRING with default parameters. (For interpretation of the references to color in this figure legend, the reader is referred to the web version of this article.)

To further support our findings at the protein level, we performed IHC on mouse, RM, and human brain tissues (Fig. 4A–C). In mice, LY6C expression was significantly lower in old brains compared to young ones (Mann–Whitney *U* test, *p-value* = 0.029). RAMP2 expression was also significantly reduced in old mice (*p-value* = 0.029) and human brains (*p-value* = 0.016) relative to young controls. In RMs, some young animals showed higher RAMP2 expression, but the difference was not statistically significant (*p-value* = 0.343). RASGRF2 protein levels were significantly higher in human brains (*p-value =* 0.016) but not in RMs (*p-value* = 0.394). Enrichment analysis identified Ras/MAPK and EGFR-associated signaling pathways linked to RASGRF2, whereas RAMP2-associated genes were enriched in adrenomedullin receptor signaling and adenylate cyclase–activating GPCR pathways (Fig. 4D and Fig. S7—A, B).

### 3.3 Age-related endothelial subtypes remodeling with preserved subtype identity

**Fig. 5:**
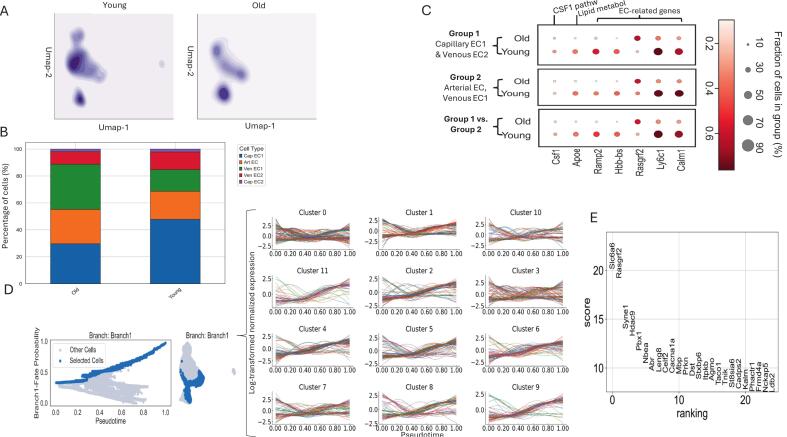
Age-related shifts in endothelial cell subtype composition without major changes in transcriptional programs. (A) UMAP plots showing the distribution of EC subtypes in young and old mice. (B) Bar plot showing subtype proportions. (C) Heat maps of aging-related genes across contracting and expanding subtypes. (D) Palantir pseudotime analysis of Branch 1 (Arterial ECs and Venous EC1), clustering 1000 randomly sampled genes. The color scale represents log-transformed normalized gene expression levels. The bold line indicates the average expression of genes in each cluster along the pseudotime trajectory. (E) Top 20 differentially expressed genes with high score ranking between young and old mice (Wilcoxon rank-sum test, *p* < 0.05). The color scale represents log-transformed normalized gene expression levels. Pseudotime = 0, root/start of the trajectory; pseudotime = 1, terminal/end of the trajectory.

We observed clear shifts in the proportions of EC subtypes between young and old mice (Fig. 5A). In the aging brain, Cap EC1 cells were reduced (45% in old vs. 57% in young), while Ven EC2 cells were markedly increased (12% vs. 2%). Art ECs also expanded (33% vs. 16%), whereas Ven EC1 cells showed only modest change (17% vs. 14%). Capillary EC2 cells remained stable at around 2% in both groups (Fig. 5B).

To determine whether these compositional changes were accompanied by transcriptional alterations, we examined the expression of genes involved in CSF1 pathway (*Csf1*) [43], lipid metabolism (*Apoe)* [44], and previously identified key EC-associated genes (*Ramp2, Hbb-bs, Rasgrf2, and Calm1*). We compared their expression patterns across expanding subtypes (Cap EC1, Ven EC2) and contracting subtypes (arterial ECs, ven EC1) but found that their expression trends were largely similar across clusters (Fig. 5C).

We next used Palantir pseudotime analysis to explore whether aging shifts transcriptional programs within EC subtypes. Six branches were defined, and we focused on Branch 1, primarily composed of arterial ECs and venous EC1 cells. By randomly sampling 1000 genes and clustering them by pseudotime trends, we observed highly similar expression patterns along this branch, reinforcing the idea that aging alters EC subtype proportions rather than their core transcriptional programs (Fig. 5D). To further explore potential age-associated transcriptional changes, we performed differential expression ranking (Wilcoxon rank sum test) between old and young ECs. The top-ranked genes included *Slc6a6* (taurine transporter TauT) and *Rasgrf2* (a Ras guanine nucleotide exchange factor), both with scores >19, followed by *Syne1, Hdac9, Pbx1*, and *Nbea* (scores >12) (Fig. 5E). Many of these genes, including *Slc6a6, Rasgrf2, Hdac9*, and *Pbx1*, have been linked to aging, endothelial cell regulation, and vascular function [54], [66], [67]. These findings suggest that aging primarily shifts EC subtype composition, while transcriptional programs remain largely conserved, with only a small set of genes showing meaningful changes.

We identified *Ramp2, Hbb-bs, Ly6c1*, and *Calm1* as common genes expressed across multiple EC subtypes, with *Rasgrf2* emerging as the only gene newly upregulated in this dataset (Fig. 2D). Both *Ly6c1* and *Calm1* are well-established markers associated with endothelial function and vascular aging [56], [68]. Based on these findings, we selected three key genes—*Ramp2, Hbb-bs*, and *Rasgrf2*—for further analysis to better understand how aging affects EC function. Using Palantir pseudotime analysis (as described earlier), we mapped gene expression dynamics along six differentiation branches representing all EC subtypes (Fig. S8A). We observed that *Hbb-bs* expression consistently decreased across all six branches (Figs. S8 B and C), while *Ramp2* showed a decline along Branch 2 (Cap EC2) and Branch 5 (Ven EC2), remaining relatively stable elsewhere. In contrast, *Rasgrf2* exhibited a strong upward trend along nearly all branches, except Branch 3 (Ven EC1). These findings indicate that the upregulation of *Rasgrf2* in aging ECs is cell–type–specific, rather than a global response, while downregulated genes such as *Ramp2* and *Hbb-bs* are also restricted to certain subsets. This highlights the importance of pinpointing the specific EC populations most affected by aging to understand endothelial dysfunction and neurovascular decline.

**Fig. 6:**
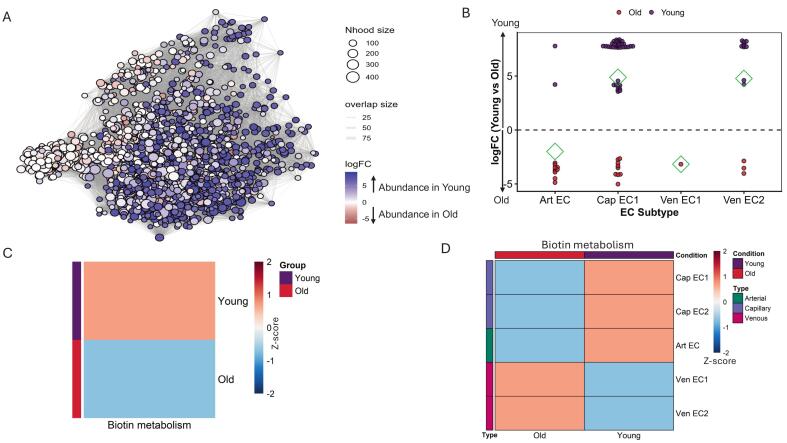
Age-associated changes in endothelial cell abundance and biotin metabolism. (A) Visualization of the neighborhood graph, where each node represents a local cell neighborhood, and node size reflects the number of cells per neighborhood (nhood size). The color scale indicates the log fold change (logFC) in abundance between young and old mice, with positive values representing enrichment in young mice and negative values indicating enrichment in old mice. Positive logFC: enriched in young; negative logFC: enriched in old. Overlapping nodes highlight neighborhoods with similar transcriptional profiles, illustrating population continuity across subtypes. (B) Summary of differential abundance results across endothelial subtypes. The green rectangle shows the average enrichment (logFC) of endothelial subtypes in young and old mice. (C—D) Factor analysis heatmap showing the expression of biotin metabolism–related factors by group (old vs. young) and endothelial cell (EC) subtypes. Column-wise z-score normalization was applied, so each column represents the relative enrichment of a SPECTRA factor across groups and subtypes. Darker colors indicate higher-than-average scores (enrichment), while lighter colors represent lower-than-average scores (depletion) relative to the mean for that factor. (For interpretation of the references to color in this figure legend, the reader is referred to the web version of this article.)

To further examine age-associated shifts in EC abundance, we applied Milo, which detects differential representation of local cell neighborhoods with a k-nearest neighbor graph. Fig. 6A shows the neighborhood graph colored by logFC between old and young mice, where each node represents a local EC neighborhood and node size reflects the number of cells within that neighborhood. Overall, a greater number of EC neighborhoods were enriched in young mice compared to old mic. Fig. 6B shows that neighborhoods corresponding to Art EC (logFC young vs old) (average logFC = −1.99) and Ven EC1 (average logFC = −3.18) are enriched in old mice (Table S4), reflecting higher representation of these subtypes in the aged samples, while neighborhoods corresponding to Cap EC1 (average logFC = 4.88) and Ven EC2 (average logFC = 4.78) are less enriched in old mice, suggesting rarefaction or cell loss with age [69]. Consistent with subtype proportion analysis, the percentages of Cap EC1 and Ven EC2 cells were reduced, whereas Ven EC1 and Art ECs were increased in the aged group. Together, these findings suggest that vascular aging is associated with a selective remodeling of EC subtypes, potentially contributing to altered vascular function in the aged brain [70].

### 3.4 Age-associated changes in biotin metabolism

We next applied Factor Analysis, a machine learning approach that identifies groups of co-varying features, to explore metabolic states, especially biotin metabolism in ECs. We focused on biotin metabolism because biotin is a key lipogenic vitamin that acts as a coenzyme for acetyl-CoA carboxylase, the rate-limiting enzyme in fatty acid biosynthesis, and is critical for maintaining brain lipid homeostasis [71]. Given the brain's high lipid content, biotin deficiency has been linked to neurological disorders [72], and emerging evidence suggests that biotin supports mitochondrial function, neuroprotection [73], [74], and potentially BBB integrity [75], making it a relevant target for studying age-related vascular and metabolic changes in ECs [76]. As a cofactor for carboxylases involved in mitochondrial fueling and lipid synthesis, biotin supports mitochondrial energy production and redox homeostasis in ECs [77]. Impairment of biotin-dependent metabolism may therefore increase oxidative stress and weaken tight junction stability, potentially compromising BBB maintenance during aging. SPECTRA identified that overall enrichment of the biotin metabolism program was less enriched in old mice compared to young mice (Fig. 6C). At the subtype level, biotin pathway enrichment was reduced in Cap EC1, and Cap EC2 in old mice, while it was more enriched in Art EC, Ven EC1 and Ven EC2 in old mice (Fig. 6D). These patterns may reflect adaptive metabolic remodeling during endothelial aging rather than uniform metabolic suppression. Together, these shifts parallel the observed changes in EC subtype proportions—Cap EC1 cells were reduced with age, Art ECs showed both increased abundance and elevated biotin pathway enrichment, suggesting that coordinated alterations in endothelial composition and metabolic state across vascular segments during aging. Notably, pathway enrichment was analyzed at the single-cell level, indicating that reduced biotin metabolism was observed within aged EC subsets and was not solely attributable to changes in subtype abundance.

### 3.5 Aging-associated remodeling of endothelial cell communication networks

**Fig. 7:**
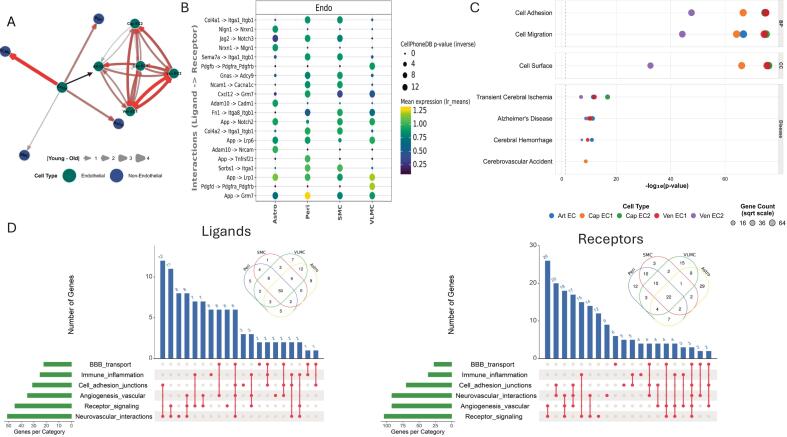
Aging alters endothelial cell communication. (A) Network maps showing interactions between endothelial cells (ECs) and non-ECs, including pericytes, smooth muscle cells (SMCs), astrocytes (Astro), and vascular leptomeningeal cells (VLMCs), as well as among EC subtypes. (B) Dot plots of the top 20 ligand-receptor interactions across EC subtypes and between ECs and other brain cells in old mice, inferred using LIANA's CellPhoneDB. (C) Differences in the number of ligand–receptor interactions between ECs and non-ECs, and among EC subtypes in old mice. (C) Enrichment analysis of ligand–receptor pairs in old mice using ToppGene. (D) Venn diagram and bar plot showing shared genes between EC–EC and EC–non-EC interactions, along with their functional annotations derived from PubMed, GeneCards, and UniProt databases.

We first assessed differences in the number of ligand–receptor interactions across conditions (Fig. 7A, B and Figs. S9). After normalization to EC numbers, aged mice exhibited increased interaction density across multiple EC subsets rather than a uniform reduction in signaling. In young mice, ligand activity was predominantly enriched in arterial ECs, whereas receptor activity was comparable across subsets. In aged brains, both ligand and receptor interaction density became more broadly distributed across capillary, venous, and arterial ECs (Table S5A). These findings indicate that aging reshapes endothelial communication architecture by redistributing signaling activity across EC subtypes rather than global communication collapse.

To account for global differences in interaction abundance between conditions, we evaluated the proportional contribution of each source–target interaction to total signaling within each age group. Overall, interaction distributions were largely preserved between young and aged mice. These results suggest that aging is associated with network-level rewiring characterized by redistribution of signaling strength rather than uniform gain or loss of endothelial communication (Table S5). Further pathway enrichment analysis of age-affected ligand–receptor pairs revealed associations with pathways related to cell adhesion, migration, and cell-surface signaling, as well as with neurovascular diseases such as cerebral ischemia, cerebral hemorrhage, and Alzheimer's disease (Fig. 7C) [24].

We next examined ligand–receptor interactions among ECs, PCs, SMCs, ACs, and VLMCs in old mice to assess whether aging reshapes EC communication networks, focusing exclusively on data from aged animals. We identified a total of 2605 significant ligand–receptor interactions between ECs and other cell types (*p*-value <0.01). Using a Venn diagram, we found 50 shared ligands and 22 shared receptors between ECs and other cell types. Many of these genes are implicated in cell–cell adhesion, receptor signaling, BBB transport, angiogenesis and vascular function, neurovascular interactions, and immune/inflammatory pathways (Fig. 7D and Table S6). For example, *ApoE, APP*, and *PSEN1* are central to amyloid processing and AD [78], while Notch signaling (*Notch2/3*) and integrins (*Itgb1/5*) are linked to vascular remodeling and BBB integrity [79], [80]. Collectively, these findings suggest that aging may alter EC communication both within the endothelial network and with other brain cell types, engaging pathways involved in BBB disruption, neuroinflammation, and Alzheimer's disease-related signaling.

## 4 Discussion

**Fig. 8:**
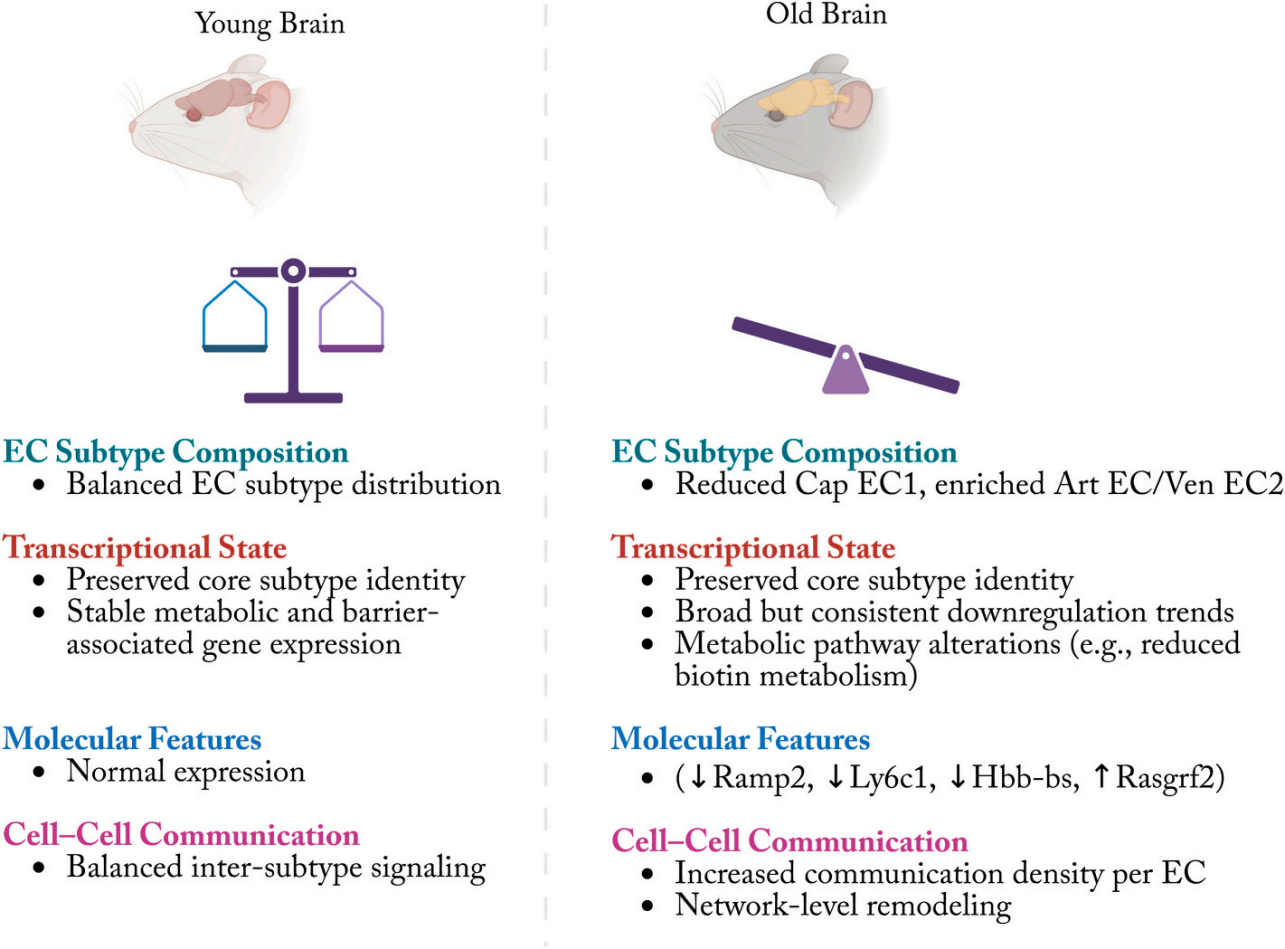
Schematic model of endothelial remodeling in the aging brain. The schematic summarizes endothelial subtype composition, molecular alterations, and communication network changes. Young brains show balanced EC subtype distribution, preserved transcriptional programs, and stable inter-subtype signaling. Aging is associated with reduced Cap EC1, relative enrichment of arterial and Ven EC2 subtypes, downregulation of Ramp2, Ly6c1, and Hbb-bs, upregulation of Rasgrf2, and redistribution of endothelial communication density across subtypes.

This study found that aging mainly shifts the relative percentage of different EC subtypes rather than causing widespread changes in gene expression across all ECs. We observed a reduction in Cap EC1 cells and an expansion of Art ECs and Ven EC2 cells. These changes suggest a remodeling of the vascular landscape, reflecting adaptation to age-related stress ([81]). This finding aligns with previous scRNA-seq studies showing segment-specific EC heterogeneity in the healthy brain [38], while Cap ECs are vulnerable in hippocampal aging models [21], [22]. The schematic model summarizing endothelial remodeling in the aging brain is shown in Fig. 8.

In our study, we identified *Ramp2, Hbb-bs, Ly6c1*, and *Calm1* as key genes consistently enriched across multiple brain EC subtypes in young mice, implying their critical roles in maintaining neurovascular integrity and their susceptibility to aging-related changes. Subtype-restricted differential expression analysis demonstrated that age-associated reductions in Ramp2, Ly6c1, and Hbb-bs occur within endothelial subtypes, indicating transcriptional downregulation rather than solely reflecting shifts in subtype composition. Consistent decreases observed in an independent dataset and corresponding reduction in capillary-associated RAMP2 and LY6C protein expression further support that these transcriptomic alterations represent biological relevant in vivo endothelial remodeling, although we acknowledge that immunostaining cannot fully resolve subtype-specific protein changes at single cell resolution.

*Ramp2* (receptor activity-modifying protein 2) is associated with calcitonin gene-related peptide signaling, which supports vasodilation and BBB integrity [53]. Its depletion in aging, particularly in Cap EC1 and EC2, likely impairs barrier function and vascular responsiveness, contributing to neurovascular decline [82]. ECs have been found to express hemoglobin α (e.g., HBA1), which interacts with eNOS to modulate vascular tone and nitric oxide signaling [83], [84]. Although the role of hemoglobin β (*Hbb-bs*, brain-specific) in ECs remains less defined, its reduced expression in aged ECs may similarly compromise the NO-dependent vasoregulatory mechanisms, potentially contributing to cerebrovascular dysfunction in the aging brain. Recent evidence indicates that endothelial redox imbalance is a central driver of cerebrovascular dysfunction during aging, with oxidative stress disrupting nitric oxide bioavailability, vascular tone regulation, and BBB integrity [85]. The age-associated downregulation of *Ramp2*, *Hbb-bs*, and *Calm1* observed in our dataset may contribute to impaired redox homeostasis, as these genes are linked to vasodilatory signaling and calcium-dependent endothelial function [86], [87]. Together, these findings suggest that transcriptional suppression of vascular maintenance genes may converge on oxidative stress pathways, amplifying endothelial vulnerability in the aged brain. Notably, oxidative stress has been causally linked to age-associated cognitive decline [88], suggesting that endothelial redox dysregulation may constitute an upstream vascular mechanism contributing to neurocognitive impairment.

*Ly6c1*, a marker of endothelial activation, regulates vascular inflammation and immune cell adhesion [89]. Its depletion indicates diminished EC-immune interactions, possibly disrupting the repair mechanism in aged vasculature. *Calm 1* (calmodulin 1), a calcium-binding protein, is essential for endothelial repair and signaling pathways like nitric oxide production ([90], p. 1). *miR-1a-3p* has been reported to suppress myosin light chain kinase signaling and exacerbate advanced glycation end products-induced endothelial barrier function impairment [61]. Decreased expression of *Calm 1*, linked to *miR-1a-3p* in our analysis, likely contributes to endothelial senescence and impaired vascular homeostasis. The consistent downregulation of these genes across EC subtypes underscores their role in sustaining EC function, and their loss in aging likely drives BBB dysfunction and increased vulnerability to neurodegenerative processes, aligning with observed capillary rarefaction and neuroinflammation [4].

In our dataset, *Rasgrf2* appeared as the only gene consistently enriched in Cap EC1 in aged mice. *Rasgrf2* encodes a guanine nucleotide exchange factor that activates Ras signaling, a pathway critical for neuronal development and synaptic plasticity [91]. In ECs, its enrichment may reflect a transcriptional response to aging-related stress, potentially enhancing neurovascular signaling to support neuronal function in the aging brain. This is consistent with its role in modulating neuronal activity [54], [55]. The selective upregulation in Cap EC1 suggests subtype-specific adaptations, possibly to counterbalance the widespread depletion of genes like *Ramp2* and *Hbb-bs* that impair vascular integrity. However, this response may be insufficient to fully mitigate age-related endothelial dysfunction, as reduced EC plasticity and BBB breakdown persist [4], highlighting *Rasgrf2* as a potential target for preserving neurovascular health [54], [55]. In functional enrichment analysis, we found that DEGs in aged brain EC subtypes are associated with SRP-dependent cotranslational targeting to the membrane, NF-κB, TNF, and p53 signaling, as well as neurodegenerative and inflammatory pathways, indicating suppressed EC function and increased senescence, consistent with previous studies on vascular aging [6], [92]. SRP-dependent cotranslational targeting refers to the process by which nascent proteins (proteins that are newly synthesized by ribosomes) are directed to the endoplasmic reticulum membrane while still in translation, ensuring proper localization and folding of membrane and secreted proteins [93], [94]. These changes, particularly in capillary ECs, contribute to BBB dysfunction and neurovascular decline [4]. This suggests aging primarily disrupts EC homeostasis, with limited compensatory responses.

In this study, we found that the expression levels of LY6C, RAMP2, and RASGRF2 are quite different across species and age groups. Species-specific differences in LY6C, RAMP2, and RASGRF2 expressions likely reflect distinct aspects of brain biology across mice, RMs, and humans. LY6C, a marker of monocytes and other immune cells, may vary with species-specific immune surveillance and recruitment patterns in the brain during aging [95]. RAMP2, a regulator of vascular integrity and signaling, could differ due to variations in cerebrovascular architecture and BBB regulation between species [96]. RASGRF2, which influences neuronal plasticity and synaptic signaling, may show divergent patterns that reflect differences in neuronal circuitry and lifespan-associated changes in cognitive resilience [97]. Altogether, these mechanistic differences underscore how aging impacts immune, vascular, and neuronal pathways in a species-dependent manner. Furthermore, enrichment analysis of *Ramp2*-associated genes highlighted adrenomedullin receptor signaling and adenylate cyclase-activating GPCR pathways, including positive regulation of protein kinase A signaling, consistent with its established role in endothelial homeostasis and vascular tone regulation. In contrast, *Rasgf2*-associated genes were enriched in Ras/MAPK and p38 MAPK pathways, which are commonly linked to stress-responsive intracellular signaling. Together, these findings suggest that aging shifts endothelial signaling balance from homeostatic GPCR-cAMP pathways toward Ras-MAPK-associated stress signaling networks.

In our brain dataset, we observed a relative decrease in cap EC1 and an increase in Art ECs with age, consistent with previous reports showing an expansion of Art ECs and a reduction of Ven ECs and Cap ECs in the aging brain [38]. This change may not necessarily indicate a global loss of capillaries but rather a shift in transcriptional states — where Art ECs expand or activate proliferative programs as a mechanism to sustain vascular function in the aging brain. The association of Art EC in old mice could reflect an attempt by these cells to compensate for age-related vascular stress or damage [98]. Normally, endothelial proliferation declines with age, but these cells may enter the cell cycle to maintain vascular integrity, even if this response is imperfect or dysregulated [99]. Enrichment for biotin metabolism in young ECs suggests that these cells require increased metabolic support for energy-demanding processes such as repair, protein synthesis, and cell cycle progression [100]. Our analysis also revealed age-associated alterations in biotin metabolism across EC subtypes. In young mice, biotin metabolism was enriched in Cap EC1, Cap EC2, and Art ECs, consistent with its role in supporting healthy EC function and metabolic homeostasis [75], [101]. In contrast, aged mice exhibited reduced biotin metabolism in these subtypes, suggesting a decline in metabolic efficiency in aging that may contribute to endothelial vulnerability [76]. Interestingly, Ven EC1 and Ven EC2 showed relatively higher biotin metabolism in aged mice, potentially representing a metabolic response aimed at preserving vascular stability and maintaining endothelial function in the face of age-related stress [74]. In young brains, ECs exhibit stable metabolic homeostasis characterized by active biotin metabolism and cap EC1-like features, supporting efficient barrier maintenance and nutrient exchange. In contrast, in old brains, ECs shift toward proliferative states, marked by arterial-like signatures, potentially reflecting a response to vascular aging and emerging BBB dysfunction. Collectively, these findings indicate that aging reshapes EC metabolic programs in a subtype-specific manner, with biotin metabolism potentially serving as a marker of both endothelial resilience and vulnerability. Because Cap ECs depend on adequate metabolic support to maintain barrier integrity and repair capacity, age-related metabolic decline may impair microvascular stability. Consistent with this, high-resolution imaging studies have demonstrated reduced capillary density and perfusion efficiency in aging brains [102], [103], [104]. These structural changes parallel the depletion of Cap EC1 in our dataset, supporting the view that capillary vulnerability is a central feature of neurovascular aging.

The observed reduction of biotin pathway enrichment in Cap ECs may have functional implications for endothelial biology during aging. Biotin-dependent metabolism supports cellular energy utilization and redox homeostasis by contributing to mitochondrial function and oxidative stress regulation [105], [106]. Because Cap ECs are the major structural and functional component of the BBB [107], metabolic remodeling in these cells may increase susceptibility to oxidative stress and impair endothelial barrier maintenance during aging. These findings suggest that age-associated vascular vulnerability may be partly driven by shifts in metabolic support pathways rather than only changes in cell abundance. Importantly, because our study is cross-sectional, we cannot determine whether biotin pathway suppression precedes capillary EC loss or occurs as a consequence of endothelial attrition. However, the persistence of reduced pathway enrichment at the single-cell level suggests intrinsic metabolic remodeling within aged Cap ECs. Future longitudinal and functional studies will be required to establish causal relationships between biotin metabolism, endothelial survival, and BBB aging.

We found that aging profoundly alters Cap EC1, affecting both cell proportions and transcriptomic profiles. This finding is consistent with previous reports showing that Cap ECs—compared with Art and Ven ECs—undergo largely transcriptional changes in normal aging [21], particularly in hippocampal endothelial cells. In our study, pseudotime analyses with Palantir further demonstrate that most core transcriptional programs in brain EC subtypes remain conserved with aging. This suggests that the fundamental molecular identity of ECs is largely preserved, even under age-related stress [108]. We found that *Ramp2, Hbb-bs, Ly6c1*, and *Calm1* were consistently downregulated across four brain EC subtypes in old mice, reflecting common aging-associated changes rather than a complete reprogramming of EC identity. We also found subtype-specific patterns, such as a high expression of *Rasgrf2* in Art EC and Ven EC2 and its low expression in Cap EC1. This combination of stable core programs and selective changes highlights that aging fine-tunes specific pathways in brain ECs, rather than broadly reshaping the entire transcriptomes.

Milo differential abundance analysis further showed fewer dynamic or transitioning neighborhoods in aged ECs, particularly Cap EC1. Because Milo captures local changes in cellular composition along the k-nearest neighbor graph, a reduction in neighborhood diversity suggests diminished endothelial plasticity with age. This loss of plasticity may underlie BBB breakdown and impaired vascular renewal, supporting observations of age-related rarefaction in capillary networks [109]. This loss of plasticity likely disrupts vascular renewal and contributes to BBB breakdown, aligning with reports of age-related capillary rarefaction [109], [110]. The weakened capacity of aged ECs to adapt and differentiate may impair their ability to maintain vascular integrity, exacerbating neurovascular decline [111]. Consistent with this interpretation, advanced imaging approaches, including functional ultrasound and ultrasound localization microscopy, have recently demonstrated age-related remodeling of the cerebral microvasculature, characterized by altered vessel density, branching patterns, and flow dynamics [112]. These structural observations align with our subtype proportion and Mio-based neighborhood analyses, which indicate selective capillary rarefaction and relative arterial enrichment. Together, molecular and imaging evidence suggest that vascular aging involves coordinated transcriptional and architectural remodeling rather than uniform endothelial decline.

We observed that aging was associated with remodeling of endothelial communication networks, characterized by a reduction in overall ligand-receptor interaction density after normalization to endothelial cell number. Together, these results suggest that aging reshapes endothelial communication networks, potentially contributing to reduced endothelial plasticity and BBB dysfunction. We also found that aging is related to disrupting EC communication, with shared ligands/receptors (e.g., *ApoE, APP, PSEN1, Notch2/3*) implicating amyloid processing, neuroinflammation, and BBB integrity pathways [113], [114]. These networks, conserved within ECs and with other brain cells, suggest amplified Alzheimer's disease-related signaling in aged brains, aligning with a study on EC-microglia interactions [115].

## 5 Limitations

In this study, we performed whole-brain analysis of ECs, which may mask region-specific effects, such as the hippocampus and frontal cortex, which may be mostly affected by aging [23], [116]. Our findings highlight Cap ECs as key sensors of aging cues, potential targets for interventions to preserve neurovascular health and reduce age-related cognitive decline. While this study offers new insight into the molecular mechanisms of EC subtypes in aging, it has several limitations. First, the small sample size (*n* = 3 mice per group) may reduce the statistical power of our analysis. Second, we focused exclusively on male mice, which limits the interpretation of potential sex-specific differences. Future studies should include female mice and incorporate spatial transcriptomics or heterochronic parabiosis to resolve heterogeneity and validate the role of circulating factors. Fourth, the number of recovered ECs between young and aged mice was unequal, which may reduce sensitivity for detecting subtle age-associated changes. Although differential expression and subtype comparisons were performed at the biological replicate level using pseudobulk aggregation, reduced endothelial cell recovery in aged samples may reflect both biological vascular remodeling and technical variability during tissue dissociation.

## 6 Conclusion

Our study provides a comprehensive single-cell transcriptomic characterization of brain ECs in young and old mice, revealing how aging reshapes EC subtype composition, transcriptional program, intercellular abundance, and cell-cell interaction. Aging leads to a substantial reduction in Cap EC1 and expansion of Art and Ven ECs, indicating a shift toward potentially dysfunctional or inflammatory states. While expression trends were largely consistent among clusters, key functional genes such as *Ramp2, Hbb-bs*, and *Calm1* decline with age, whereas *Rasgrf2* becomes selectively enriched, suggesting subtype-specific transcriptional remodeling associated with aging. Aging also disrupts intracellular abundance, alters biotin metabolism, and age-associated remodeling of ligand-receptor signaling, engaging pathways linked to neurovascular decline, inflammation, and neurodegeneration. Together, these findings highlight that age-associated endothelial dysfunction is driven more by shifts in subtype abundance and neighborhood interactions than by reprogramming of cell states. This work provides a foundational resource for understanding EC aging and identifies potential targets to preserve BBB integrity and neurovascular health in the aging brain.

## CRediT authorship contribution statement

**Hai Duc Nguyen:** Writing – original draft, Visualization, Methodology, Investigation, Formal analysis, Data curation. **Summer Siddiqui:** Writing – review & editing, Visualization, Validation, Investigation. **Diana G. Bohannon:** Writing – review & editing, Methodology, Investigation. **Robert V. Blair:** Visualization, Methodology. **Hong-Wen Deng:** Writing – review & editing. **Alexandre Prat:** Writing – review & editing, Conceptualization. **Woong-Ki Kim:** Writing – review & editing, Writing – original draft, Visualization, Validation, Supervision, Software, Resources, Project administration, Methodology, Investigation, Funding acquisition, Formal analysis, Data curation, Conceptualization.

## Author Agreement

All authors agree to submit this manuscript to the Journal and understand that they will be accountable for all aspects of the work.

## Declaration of competing interest

The authors declare no competing interests.

## Data Availability

Data will be available from the corresponding author upon reasonable request.

## Appendix A Supplementary data

Supplementary material 1 **Table S1.** Differential expression profiles of the current study and a previous study (GEO accession number: GSE147693): FDR-adjusted *P*-value <0.05 and an absolute value of log2FC exceeding 0.25. **Table S2.** 57 shared differential expression profiles of the current study and a previous study (GEO accession number: GSE147693). **Table S3.** Role of shared differential expression profiles of the current study and a previous study (GEO accession number: GSE147693). **Table S4.** Intracellular abundance of brain endothelial cell subsets in old vs young mice (SpatialFDR<0.05). logFC>0, abundance in old; logFC<0, abundance in young. **Table S5.** Differences in ligand and receptors across endothelial subsets in young and old mice. **Table S6.** Role of ligands and receptors implicated in BBB integrity.

Supplementary material 2 **Fig. S1. Quality control of scRNA-seq dataset.** (A) Histogram of Scrublet doublet scores across clusters, showing no outliers (resolution = 0.5), indicating minimal doublet contamination. (B) UMAP plot post-Scanorama batch correction, demonstrating even distribution of young and old mouse samples across clusters, confirming minimal batch effects. **Fig. S2. Cell cycle distribution in young and old mice.** (A) UMAP plots showing cell cycle phase assignments (G1, S, G2/M) in endothelial cells. Bar plots showing cell cycle phase assignments (G1, S, G2/M) for each sample (B) and EC subtype (C), generated using Scanpy's tl.score_genes_cell_cycle. **Fig. S3. Validation of endothelial cell identity.** (A) Violin plots confirming high expression of *Pecam1*, *Cdh5*, and *Slc2a1* in the “333 Endo NN” EC cluster. The color scale represents log-transformed normalized gene expression levels. (B) Expression patterns of canonical genes related to EC subtypes, pan ECs, and lymphatic ECs. (C) SEACells metacell box plot, with >95% alignment to the EC cluster, highlighting distinct EC states. **Fig. S4. Differential expression analysis details.** Volcano plots of DEGs (adjusted *p* < 0.05, |log2FC| ≥ 1) for each EC subtype, showing predominantly depleted genes in old mice and *Rasgrf2* as the primary enriched gene. NS: non-significant. **Fig. S5. Expression of Ramp2, Hbb-bs, and Rasgrf2 details.** Violin plots of Ramp2 and Hbb-bs, and Rasgrf2 by condition and each sample. The color scale represents log-transformed normalized gene expression levels. **Fig. S6. Enrichment analysis, network interaction between genes, miRNAs, and transcription factors insights into capillary EC1 DEGs.** (A–B) Enrichment plots of pathways (e.g., NF-κB, TNF, neurodegeneration) for Capillary EC1 DEGs. (C) Network plot linking *Calm1* to miR-1a-3p and *Cdkn1a* to miR-294-3p, miR-295-3p, miR-302d-3p, miR-291b-3p. (D) Transcription factor network *TP53*, *EGR1*, *ATF3*, and *MYC* regulating Capillary EC1 DEGs. **Fig. S7.** STRING-based enrichment analysis of RASGRF2 (A) and RAMP2 (B). Protein–protein interaction enrichment analysis was generated using STRING with default parameters. **Fig. S8. Subtype-specific gene expression dynamics in aging endothelial cells** (A) Palantir pseudotime trajectories across six differentiation branches representing all EC subtypes. (B) Line plots showing gene expression trends along branches: Hbb-bs is consistently downregulated, Ramp2 decreases in Branch 2 (Capillary EC2) and Branch 5 (Venous EC2), and Rasgrf2 is upregulated across most branches except Branch 3 (Venous EC1). The color scale represents log-transformed normalized expression. Pseudotime = 0, root/start of the trajectory; pseudotime = 1, terminal/end of the trajectory. (C) Heatmaps of gene expression along pseudotime. Expression is visualized as a smoothed, log1p-normalized heatmap, with values averaged across cells in each pseudotime bin. **Fig. S9.** Cell-cell interaction between endothelial cells (ECs), non-ECs, and EC subsets in young and old mice. (A) Network interactions between ECs and non-ECs, including pericytes, smooth muscle cells (SMCs), astrocytes (Astro), and vascular leptomeningeal cells (VLMCs). Chord diagram shows interactions between ECs, pericytes, SMCs, and VLMCs. Each sector represents a cell type, and the connecting chords indicate ligand-receptor interactions. The thickness of each chord corresponds to the number of interactions between the connected cell types.
