# Supplementary material for: Single-cell transcriptomic analysis reveals age-related remodeling of brain endothelial cells
Source: Aging Brain. 2026 Jul 31;10:100164. doi: 10.1016/j.nbas.2026.100164 (PMC13452397; doi:10.1016/j.nbas.2026.100164)

Figure S1

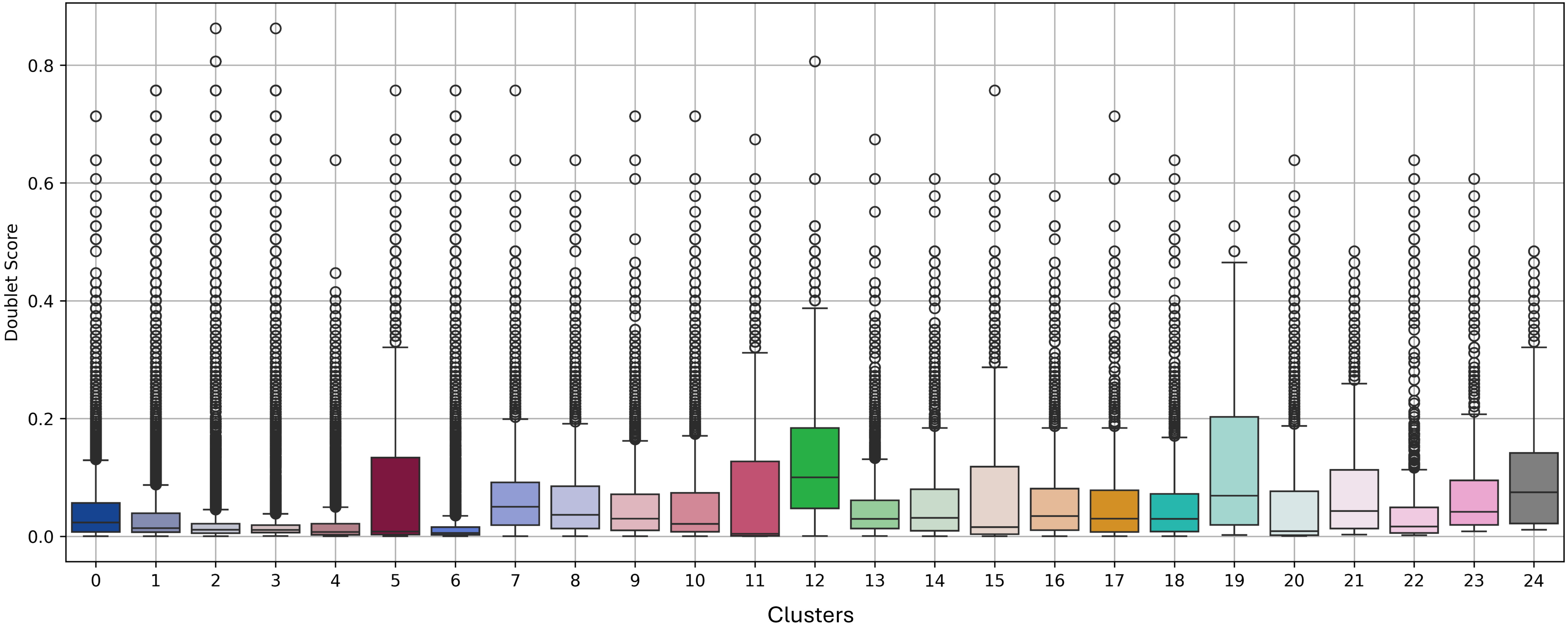
Doublet Score
0.0
0.2
0.4
0.6
0.8
0 1 2 3 4 5 6 7 8 9 10 11 12 13 14 15 16 17 18 19 20 21 22 23 24
Clusters

Figure S2

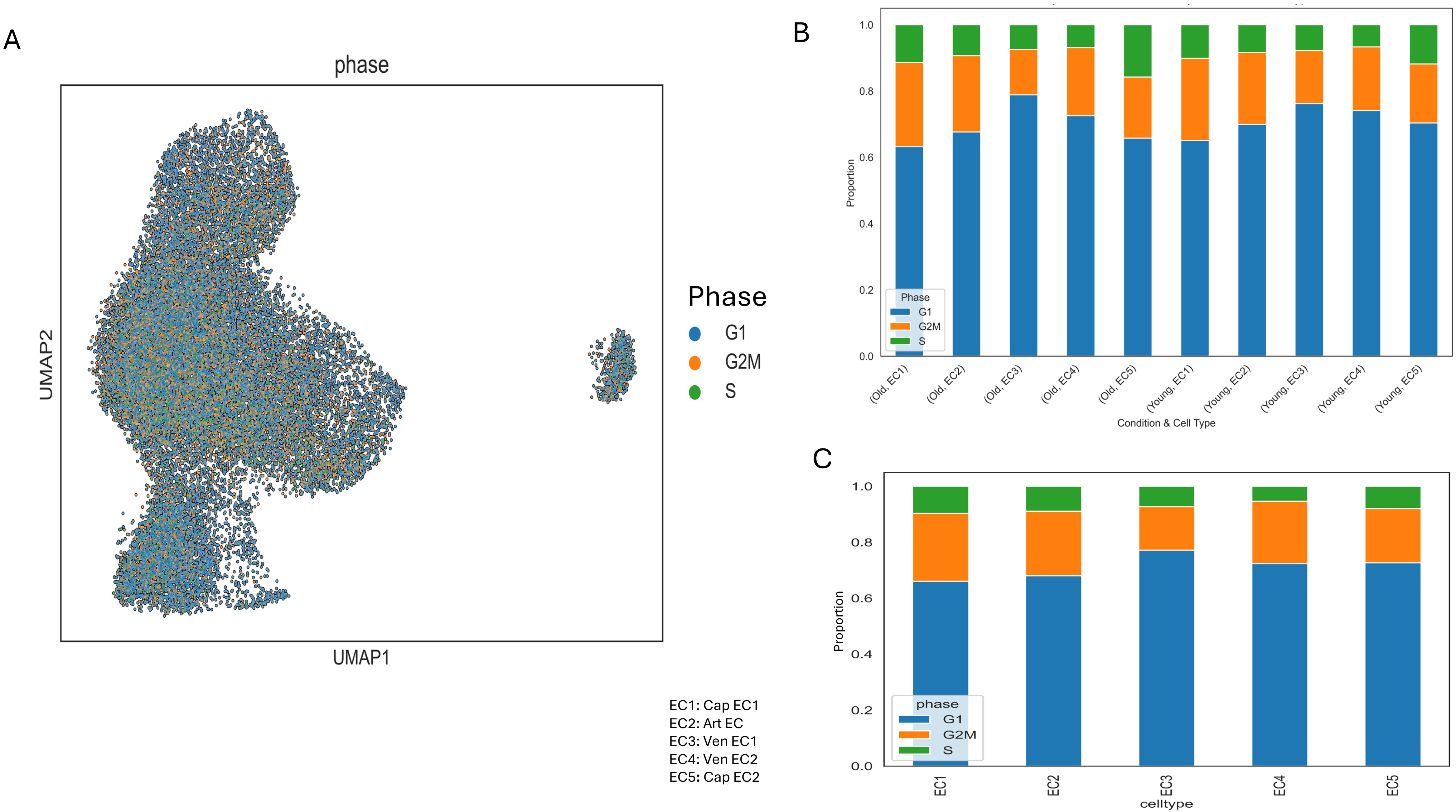

EC1: Cap EC1
EC2: Art EC
EC3: Ven EC1
EC4: Ven EC2
EC5: Cap EC2

Figure S3

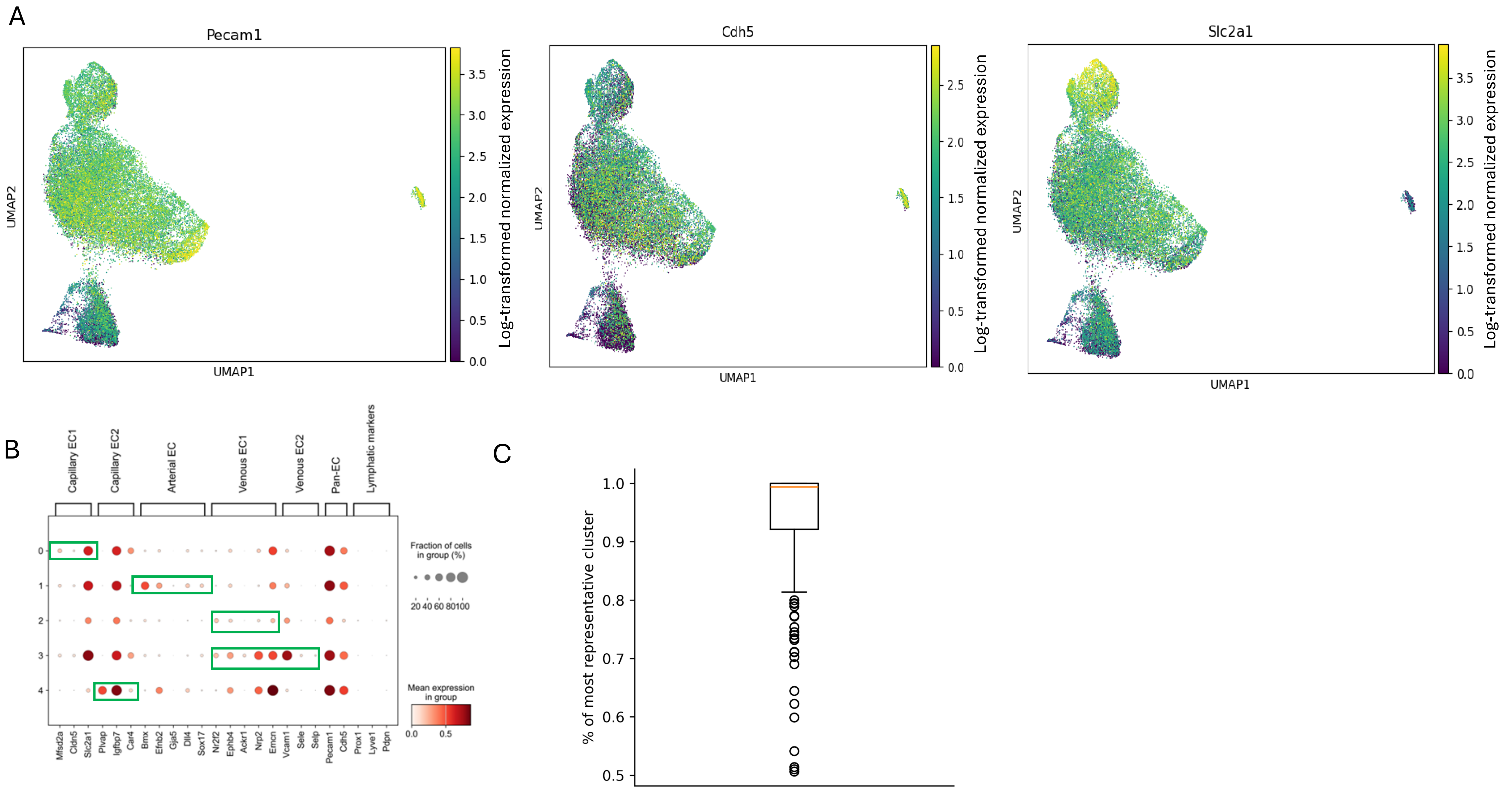
A
Pecam1
Cdh5
Slc2a1
UMAP1
UMAP2
Log-transformed normalized expression
B
Capillary EC1
Capillary EC2
Arterial EC
Venous EC1
Venous EC2
Pan-EC
Lymphatic markers
Mfsd2a
Cldn5
Slc2a1
Plvap
Igfbp7
Car4
Bmx
Efnb2
Gja5
Dll4
Sox17
Nr2f2
Ephb4
Ackr1
Nrp2
Emcn
Vcam1
Sele
Selp
Pecam1
Cdh5
Prox1
Lyve1
Pdpn
Fraction of cells in group (%)
20 40 60 80 100
Mean expression in group
0.0 0.5
C
% of most representative cluster

Figure S4

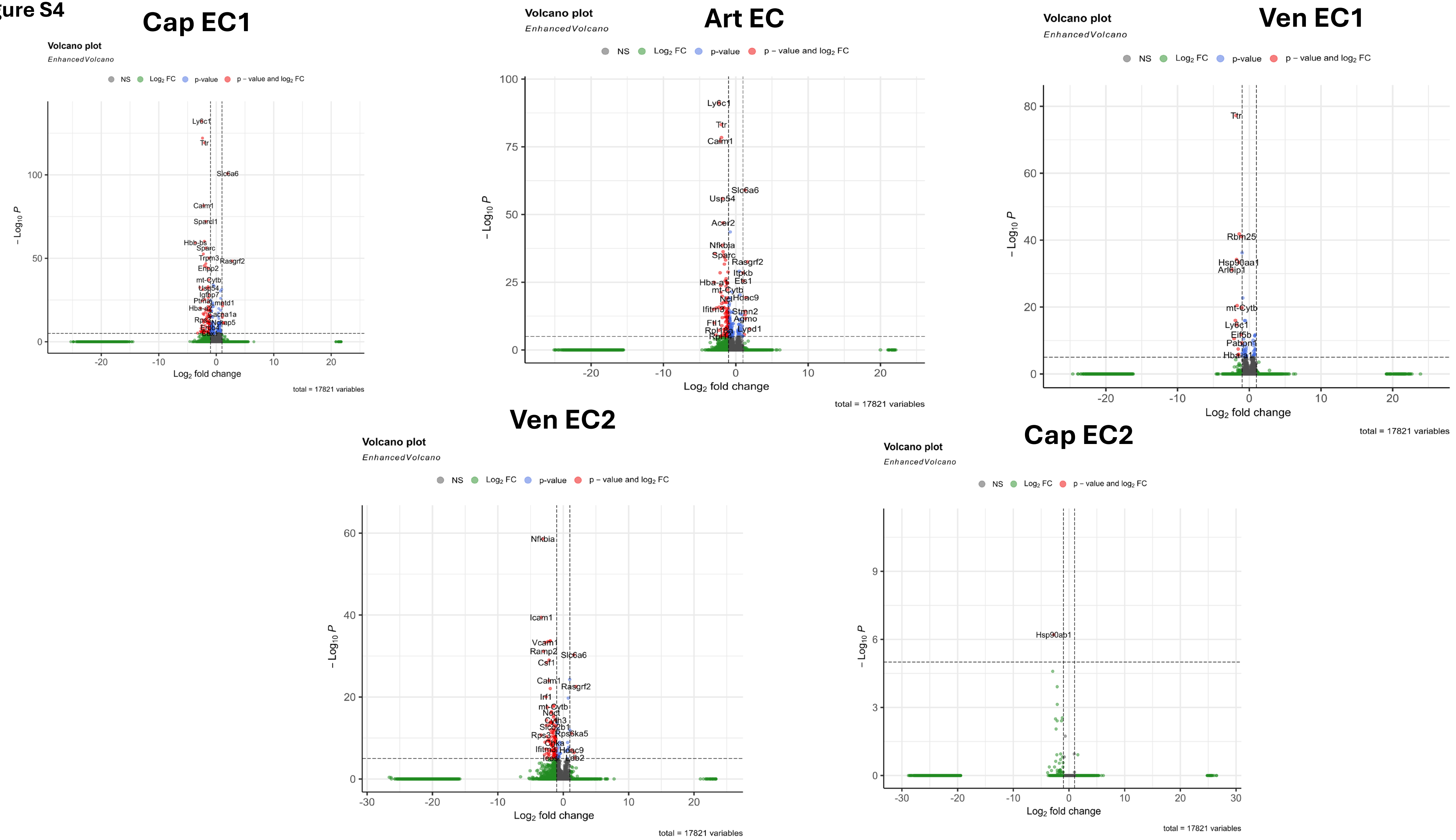

Cap EC1
Volcano plot
EnhancedVolcano
NS
Log2 FC
p-value
p – value and log2 FC
Ly6c1
Ttr
Slc6a6
Calm1
Sparcl1
Hbb-bs
Sparc
Trpm3
Rasgrf2
Enpp2
mt-Cytb
Usp54
Igfbp7
Ptma
Lmntd1
Hba-a1
Cacna1a
Nckap5
– Log10 P
Log2 fold change
total = 17821 variables
Art EC
Volcano plot
EnhancedVolcano
NS
Log2 FC
p-value
p – value and log2 FC
Ly6c1
Ttr
Calm1
Slc6a6
Usp54
Acer2
Nfkbia
Sparc
Rasgrf2
Itpkb
Ets1
Hba-a1
mt-Cytb
Hdac9
Ifitm3
Stmn2
Agmo
Ftl1
Lypd1
– Log10 P
Log2 fold change
total = 17821 variables
Ven EC1
Volcano plot
EnhancedVolcano
NS
Log2 FC
p-value
p – value and log2 FC
Ttr
Rbm25
Hsp90aa1
Arl6ip1
mt-Cytb
Ly6c1
Eif5b
Pabpn1
Hba-a1
– Log10 P
Log2 fold change
total = 17821 variables
Ven EC2
Volcano plot
EnhancedVolcano
NS
Log2 FC
p-value
p – value and log2 FC
Nfkbia
Icam1
Vcam1
Ramp2
Slc6a6
Csf1
Calm1
Rasgrf2
Irf1
mt-Cytb
Rps6ka5
Ifitm3
Hdac9
Id2
– Log10 P
Log2 fold change
total = 17821 variables
Cap EC2
Volcano plot
EnhancedVolcano
NS
Log2 FC
p – value and log2 FC
Hsp90ab1
– Log10 P
Log2 fold change
total = 17821 variables

Figure S5

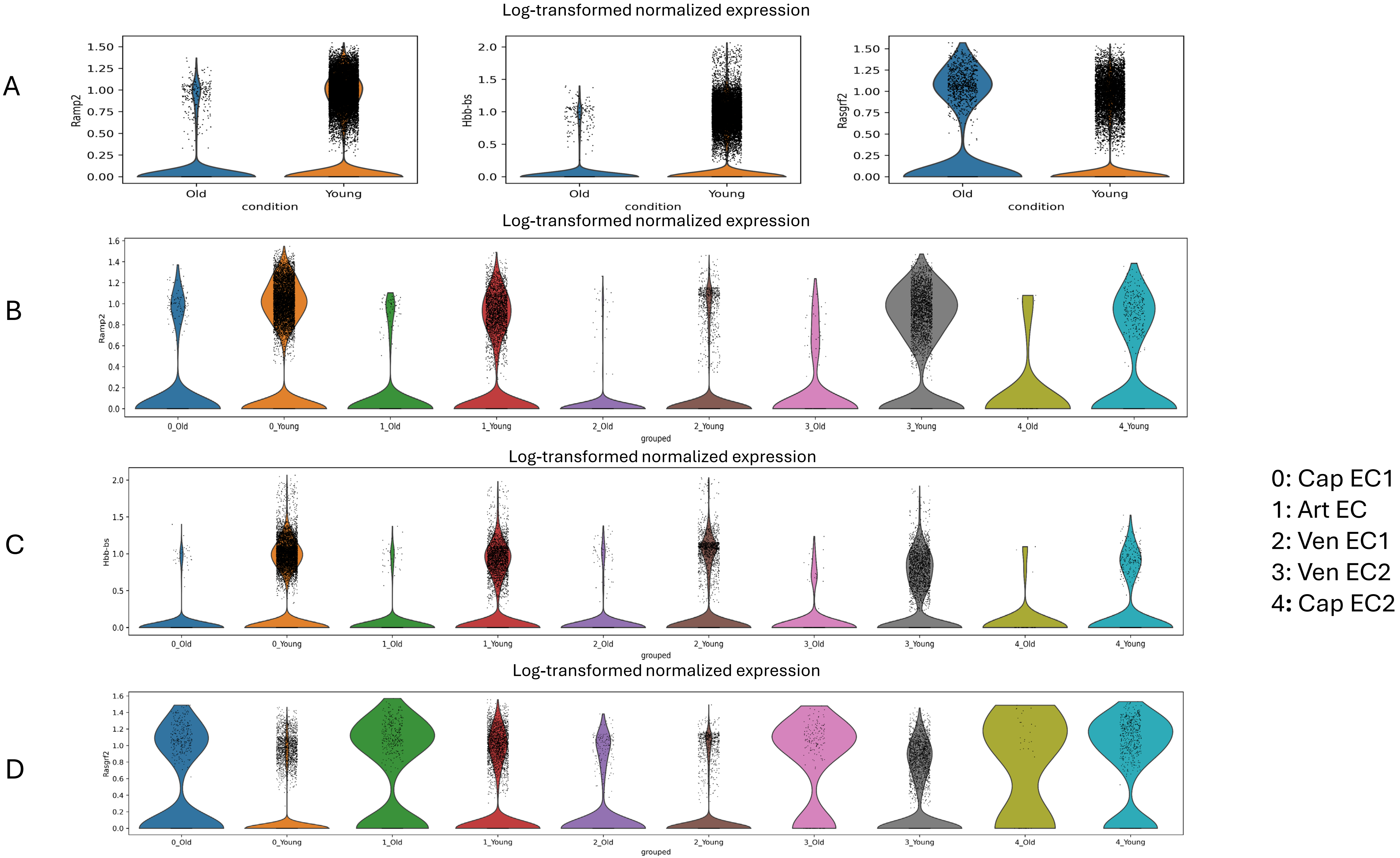
Log-transformed normalized expression
A
Ramp2
Hbb-bs
Rasgrf2
Old
Young
condition
Log-transformed normalized expression
B
Ramp2
0_Old
0_Young
1_Old
1_Young
2_Old
2_Young
3_Old
3_Young
4_Old
4_Young
grouped
Log-transformed normalized expression
C
Hbb-bs
grouped
Log-transformed normalized expression
D
Rasgrf2
grouped
0: Cap EC1
1: Art EC
2: Ven EC1
3: Ven EC2
4: Cap EC2

Figure S6

A

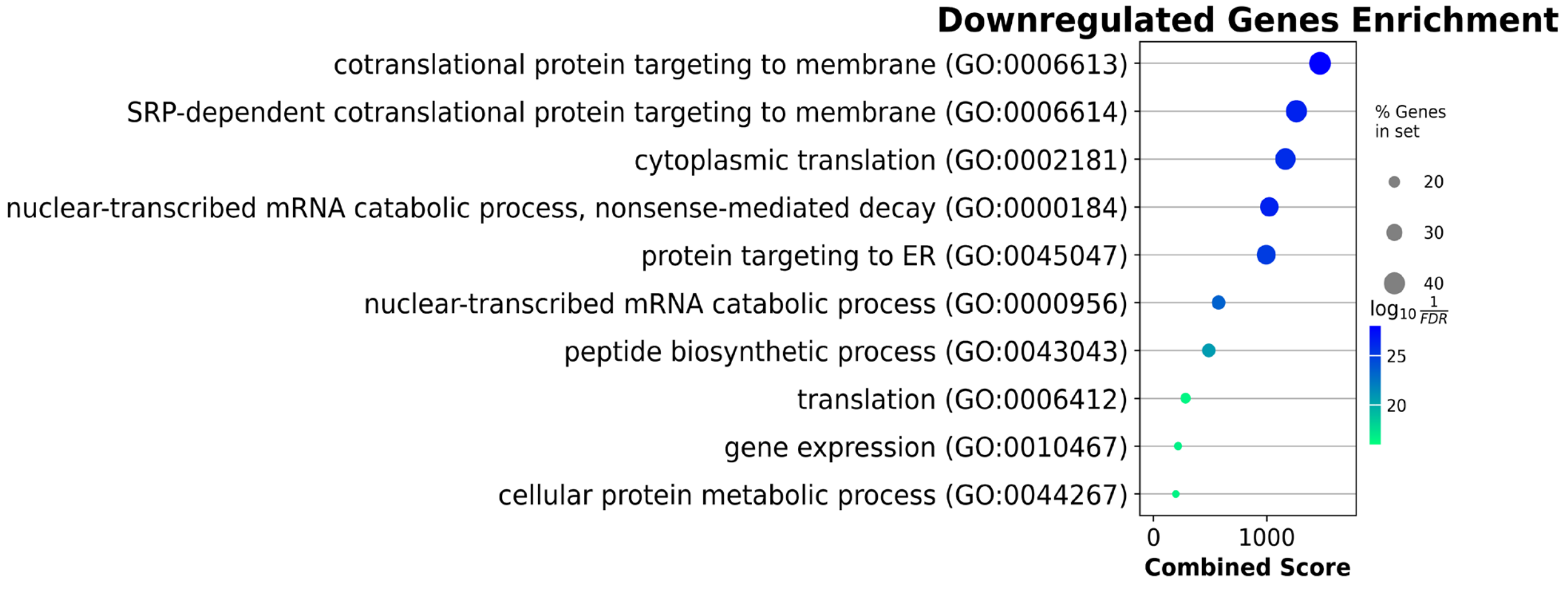

B

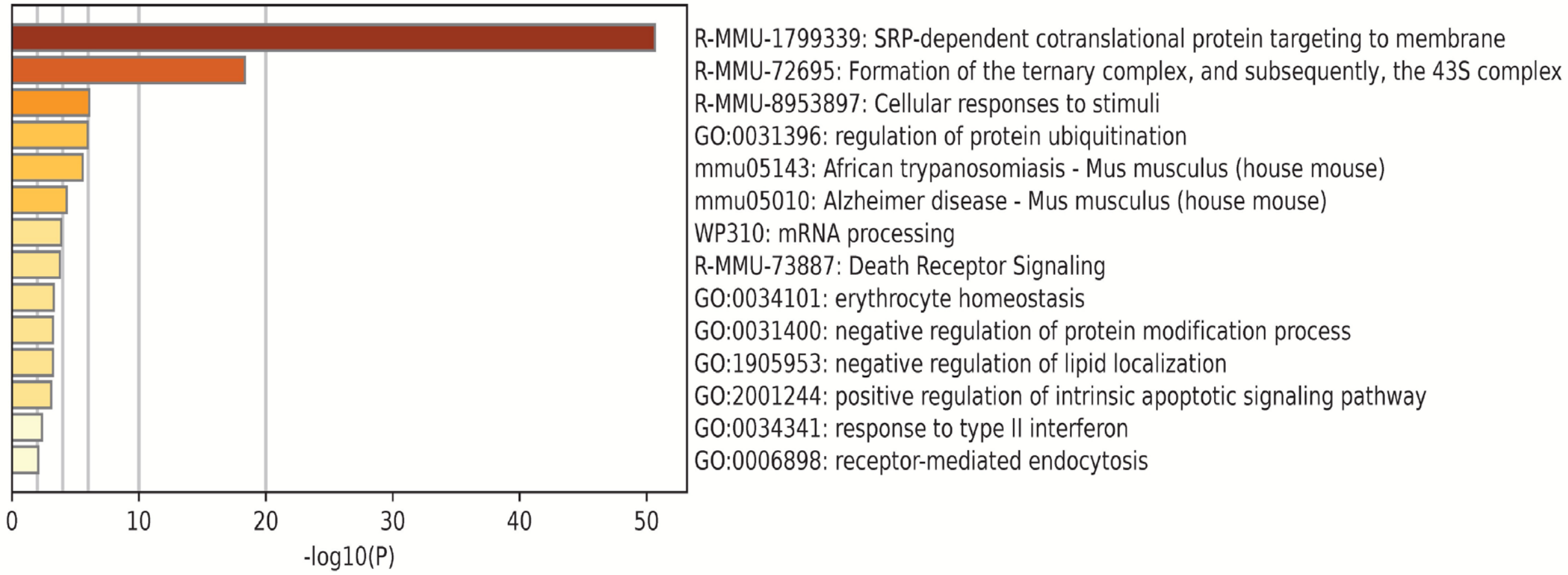

C

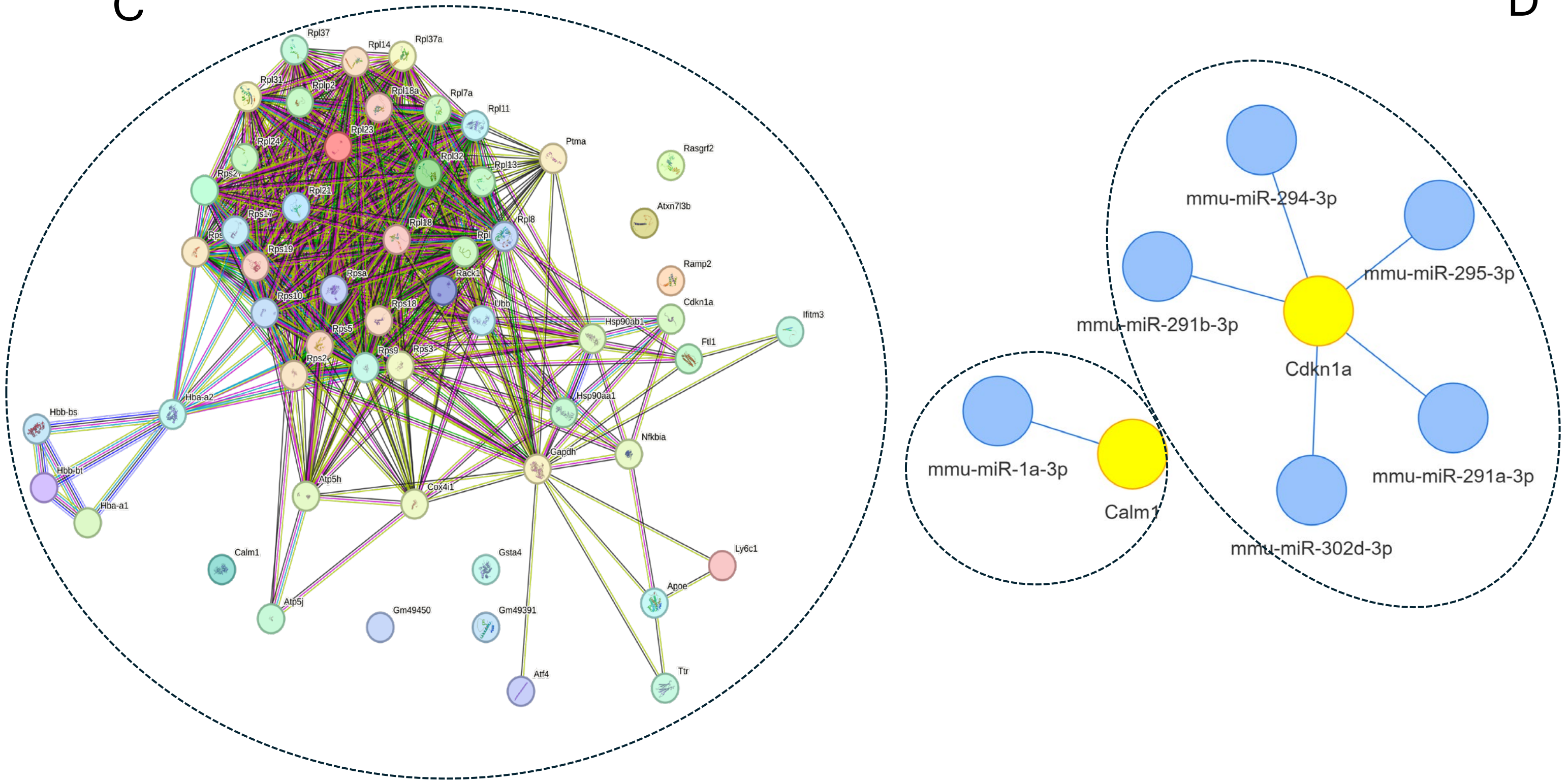

D

Rank	TF	Mean Rank	Overlapping Genes	Library
1	ZNF581	19.33	24	ARCHS4 Coexpression,1;Enrichr Queries,10;GTEx Coexpression,47
2	POU5F1B	27.0	19	ARCHS4 Coexpression,47;GTEx Coexpression,7
3	TP53	33.2	30	Literature ChIP-seq,56;ARCHS4 Coexpression,7;Enrichr Queries,55;ReMap ChIP-seq,44;GTEx Coexpression,4
4	PA2G4	48.0	12	ARCHS4 Coexpression,10;GTEx Coexpression,86
5	ZNF598	59.67	13	ARCHS4 Coexpression,121;Enrichr Queries,32;GTEx Coexpression,26
6	ZBED6	66.0	5	ARCHS4 Coexpression,66
7	EGR1	66.67	32	Literature ChIP-seq,76;ARCHS4 Coexpression,21;ENCODE ChIP-seq,40;Enrichr Queries,98;ReMap ChIP-seq,64;GTEx Coexpression,101
8	CENPA	67.0	7	ARCHS4 Coexpression,51;GTEx Coexpression,83
9	ATF3	71.0	32	Literature ChIP-seq,39;ARCHS4 Coexpression,143;ENCODE ChIP-seq,9;Enrichr Queries,85;ReMap ChIP-seq,80;GTEx Coexpression,70
10	MYC	73.17	38	Literature ChIP-seq,1;ARCHS4 Coexpression,334;ENCODE ChIP-seq,6;Enrichr Queries,45;ReMap ChIP-seq,26;GTEx Coexpression,27

Figure S7

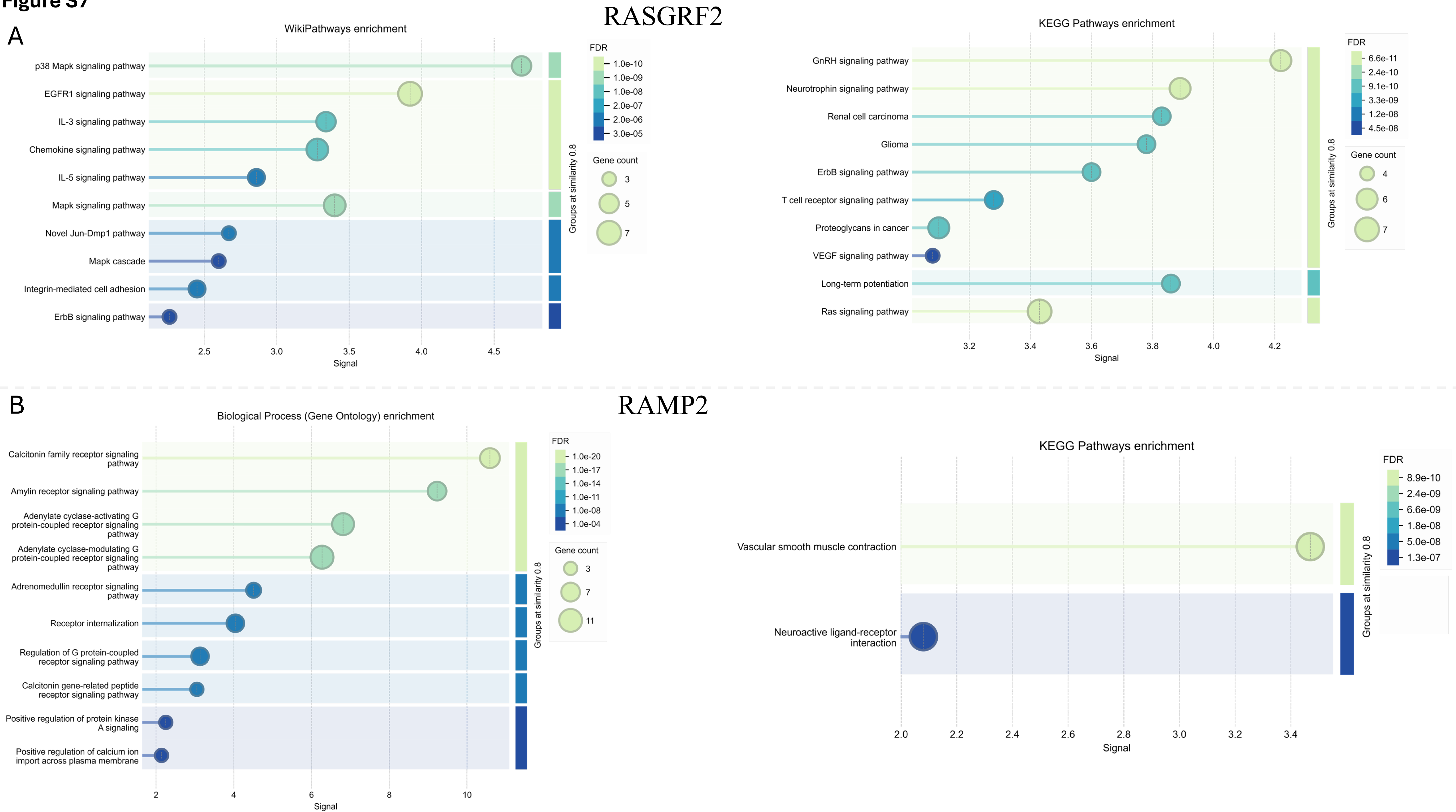

A
RASGRF2
WikiPathways enrichment
p38 Mapk signaling pathway
EGFR1 signaling pathway
IL-3 signaling pathway
Chemokine signaling pathway
IL-5 signaling pathway
Mapk signaling pathway
Novel Jun-Dmp1 pathway
Mapk cascade
Integrin-mediated cell adhesion
ErbB signaling pathway
2.5 3.0 3.5 4.0 4.5
Signal
Groups at similarity 0.8
FDR
1.0e-10
1.0e-09
1.0e-08
2.0e-07
2.0e-06
3.0e-05
Gene count
3
5
7
KEGG Pathways enrichment
GnRH signaling pathway
Neurotrophin signaling pathway
Renal cell carcinoma
Glioma
ErbB signaling pathway
T cell receptor signaling pathway
Proteoglycans in cancer
VEGF signaling pathway
Long-term potentiation
Ras signaling pathway
3.2 3.4 3.6 3.8 4.0 4.2
Signal
Groups at similarity 0.8
FDR
6.6e-11
2.4e-10
9.1e-10
3.3e-09
1.2e-08
4.5e-08
Gene count
4
6
7
B
RAMP2
Biological Process (Gene Ontology) enrichment
Calcitonin family receptor signaling pathway
Amylin receptor signaling pathway
Adenylate cyclase-activating G protein-coupled receptor signaling pathway
Adenylate cyclase-modulating G protein-coupled receptor signaling pathway
Adrenomedullin receptor signaling pathway
Receptor internalization
Regulation of G protein-coupled receptor signaling pathway
Calcitonin gene-related peptide receptor signaling pathway
Positive regulation of protein kinase A signaling
Positive regulation of calcium ion import across plasma membrane
2 4 6 8 10
Signal
Groups at similarity 0.8
FDR
1.0e-20
1.0e-17
1.0e-14
1.0e-11
1.0e-08
1.0e-04
Gene count
3
7
11
KEGG Pathways enrichment
Vascular smooth muscle contraction
Neuroactive ligand-receptor interaction
2.0 2.2 2.4 2.6 2.8 3.0 3.2 3.4
Signal
Groups at similarity 0.8
FDR
8.9e-10
2.4e-09
6.6e-09
1.8e-08
5.0e-08
1.3e-07

Figure S8

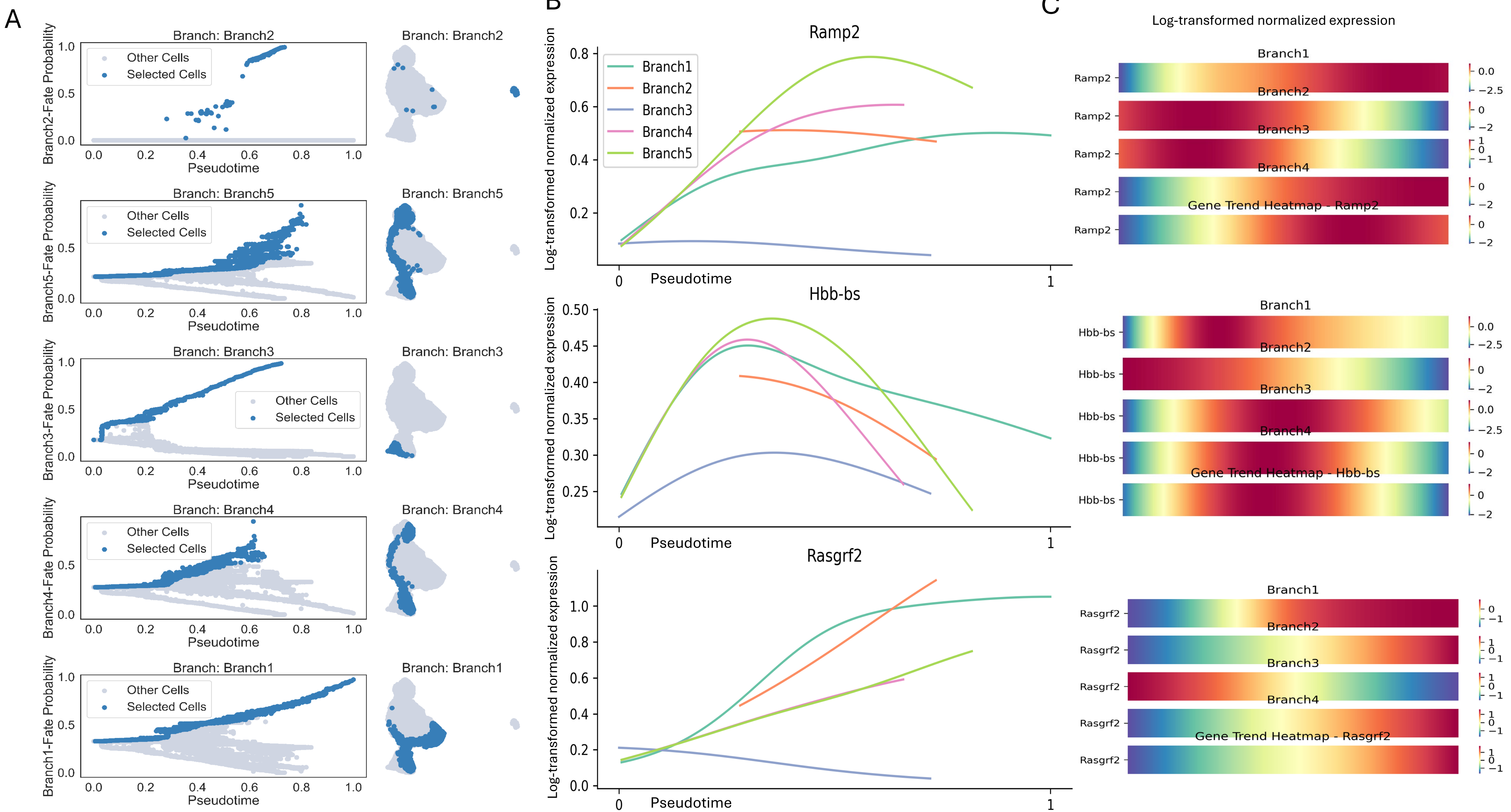
A
B
C
Branch: Branch2
Branch2-Fate Probability
Other Cells
Selected Cells
Pseudotime
Branch: Branch5
Branch5-Fate Probability
Branch: Branch3
Branch3-Fate Probability
Branch: Branch4
Branch4-Fate Probability
Branch: Branch1
Branch1-Fate Probability
Ramp2
Hbb-bs
Rasgrf2
Log-transformed normalized expression
Branch1
Branch2
Branch3
Branch4
Branch5
Gene Trend Heatmap - Ramp2
Gene Trend Heatmap - Hbb-bs
Gene Trend Heatmap - Rasgrf2

Figure S9

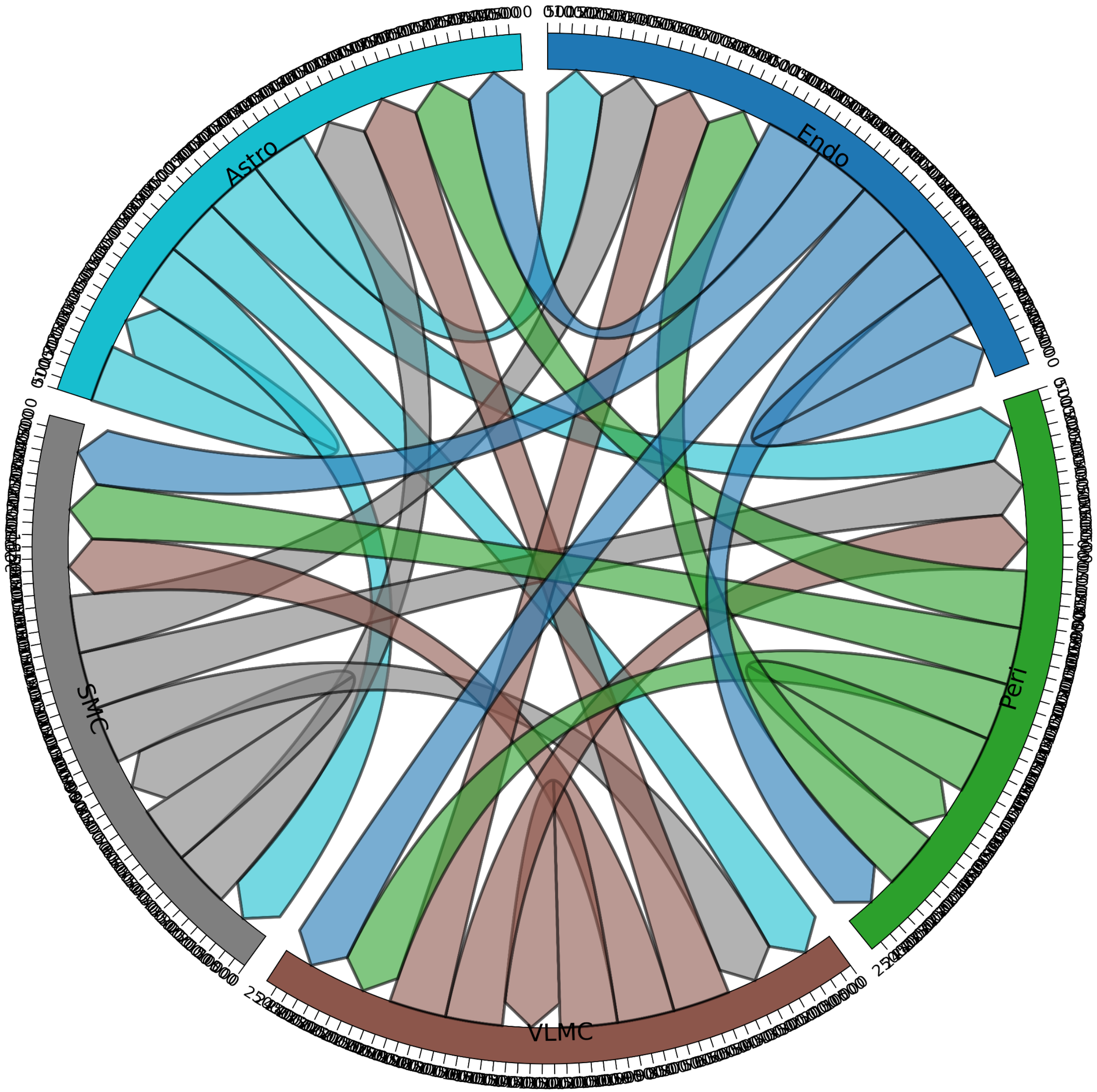

Supplement: Supplementary file 1 [file mmc1.pdf]
